# Evidence for a Golgi-to-Endosome Protein Sorting Pathway in *Plasmodium falciparum*

**DOI:** 10.1371/journal.pone.0089771

**Published:** 2014-02-25

**Authors:** Priscilla Krai, Seema Dalal, Michael Klemba

**Affiliations:** Department of Biochemistry, Virginia Tech, Blacksburg, Virginia, United States of America; Johns Hopkins Bloomberg School of Public Health, United States of America

## Abstract

During the asexual intraerythrocytic stage, the malaria parasite *Plasmodium falciparum* must traffic newly-synthesized proteins to a broad array of destinations within and beyond the parasite's plasma membrane. In this study, we have localized two well-conserved protein components of eukaryotic endosomes, the retromer complex and the small GTPase Rab7, to define a previously-undescribed endosomal compartment in *P. falciparum*. Retromer and Rab7 co-localized to a small number of punctate structures within parasites. These structures, which we refer to as endosomes, lie in close proximity to the Golgi apparatus and, like the Golgi apparatus, are inherited by daughter merozoites. However, the endosome is clearly distinct from the Golgi apparatus as neither retromer nor Rab7 redistributed to the endoplasmic reticulum upon brefeldin A treatment. Nascent rhoptries (specialized secretory organelles required for invasion) developed adjacent to endosomes, an observation that suggests a role for the endosome in rhoptry biogenesis. A *P. falciparum* homolog of the sortilin family of protein sorting receptors (PfSortilin) was localized to the Golgi apparatus. Together, these results elaborate a putative Golgi-to-endosome protein sorting pathway in asexual blood stage parasites and suggest that one role of retromer is to mediate the retrograde transport of PfSortilin from the endosome to the Golgi apparatus.

## Introduction

The human malaria parasite *Plasmodium falciparum* is responsible for approximately one million deaths annually [1]. The pathology of malaria is caused by infection of the host's erythrocytes. Within the erythrocyte, the parasite undergoes a ∼48 hour replication cycle, generating 8–26 daughter merozoites that egress from the spent host cell and invade fresh erythrocytes [2]. During this cycle, the parasite must replicate its heritable organelles (the nucleus, endoplasmic reticulum, Golgi apparatus, mitochondrion and apicoplast) and generate others *de novo*. Of the latter group, the food vacuole, rhoptries, micronemes, and dense granules are the best characterized [3], [4] but other compartments (exonemes and mononemes) have also been reported [5], [6]. The food vacuole is an acidic organelle in which endocytosed host cell hemoglobin is degraded to amino acids; it is also the site of action of quinoline anti-malarials such as chloroquine. Rhoptries, micronemes and dense granules are specialized, apically oriented secretory organelles that discharge their contents during and shortly after host cell invasion.

Intraerythrocytic *P. falciparum* must accurately sort and traffic newly-synthesized proteins to all of these intracellular organelles, several of which are not present in well-studied eukaryotic model organisms. In addition to intracellular protein trafficking, the parasite exports endogenous proteins beyond its plasma membrane, first into the parasitophorous vacuole and then in some cases into the host cell [7]. There is abundant evidence that the parasite relies heavily on its endomembrane (or secretory) system to sort and traffic both intracellular and extracellular proteins to their proper destinations (recently reviewed in [8]). Many proteins targeted to the food vacuole, apicoplast, rhoptries, micronemes, and dense granules possess a canonical “signal peptide”, a short sequence of hydrophobic amino acids near the amino terminus of the protein, that specifies co-translational import into the endoplasmic reticulum (ER). In intraerythrocytic *P. falciparum*, the ER is largely perinuclear with a couple of “horn-like” projections [9]. From the ER, most proteins appear to be transported to the parasite Golgi apparatus (the exceptions being apicoplast-targeted proteins, which bypass the Golgi [10]). These conclusions are largely drawn from experiments that demonstrate the inhibition of trafficking by brefeldin A, a fungal metabolite that blocks anterograde ER-to-Golgi traffic and causes Golgi proteins to redistribute to the ER [11], [12].

There have been conflicting reports regarding the nature of the Golgi apparatus in blood-stage *P. falciparum*. In many eukaryotic organisms, the Golgi apparatus is composed of several biochemically distinct compartments called cisternae (designated *cis*-, medial-, and *trans*-). These cisternae are closely apposed or “stacked” in mammalian cells but may also exist as unstacked cisternae as is the case in *Saccharomyces cerevisiae* [13]. Localization studies using antibodies against *P. falciparum* homologs of proteins that reside in the *cis*-Golgi (ERD2 and Bet3p) and the *trans*-Golgi (Rab6) cisternae of mammalian cells have suggested that the Golgi apparatus in *P. falciparum* is composed of dispersed, unstacked *cis*- and *trans*-cisternae [14], [15]. In contrast, localization of fluorescent protein-tagged versions of the *cis*-Golgi marker PfGRASP and the *trans*-Golgi marker PfRab6 in live cells has indicated that these two proteins are in close proximity [16], although whether they label the same or different compartments has not yet been fully resolved. Numerous ultrastructural studies of intraerythrocytic parasites, including those that have employed serial sectioning and three dimensional reconstruction, have failed to detect stacked Golgi cisternae in the parasite [17]–[19], which raises the possibility that the plasmodial Golgi apparatus is a “stripped-down” form of the organelle consisting of a single compartment. It has been speculated that the absence of N-linked glycosylation in *P. falciparum* [20] renders unnecessary the presence of biochemically distinct Golgi compartments [21].

The multi-compartment endosomal network is a key constituent of the endomembrane system. The mammalian endosomal network consists of discrete compartments (early, late and recycling endosomes) that collectively serve as a “hub” of cellular protein traffic [22]. Early endosomes are generated from endocytic vesicles and are characterized by the presence of phosphatidylinositol-3-phosphate (PI3P) and the small GTPase Rab5 at the cytosolic leaflet of the membrane. Early endosomes undergo a process of maturation that involves the replacement of Rab5 with Rab7 and the conversion of PI3P to phosphatidylinositol-3,5-bisphosphate [23]. Biosynthetic traffic intersects with the endosomal network during the transition from early to late endosomes. Late endosomes ultimately fuse with lysosomes, delivering their biosynthetic cargo to its final destination. This endosomal maturation pathway is also used to deliver membrane proteins (*e.g.* signaling receptors) from the plasma membrane to the lysosome for degradation. Some membrane proteins avoid degradation and are cycled back to the plasma membrane *via* so-called recycling endosomes. The nature of the endosomal network in *P. falciparum* has, to our knowledge, not yet been investigated. Structures resembling the multi-vesicular bodies of mammalian endosomes have been observed in parasites expressing a dominant negative mutant of the GTPase Vps4 [24]; however, it is not clear whether these structures are present in wild-type parasites.

The aim of this study was first to define endosomal compartments in intraerythrocytic *P. falciparum* and then to interrogate their contribution to protein sorting and trafficking. We focused our investigation on two highly conserved species found on the cytosolic leaflet of the endosomal membrane: the retromer cargo-selective complex and the small GTPase Rab7. The retromer cargo-selective complex is comprised of three proteins, termed Vps26, Vps29 and Vps35, which associate into a stable trimeric assembly [25]. The retromer cargo-selective complex is recruited to the mammalian endosomal membrane by prenylated, GTP-bound Rab7 [26], [27]. One role of retromer that is conserved from yeast to mammalian cells is the recycling of protein sorting receptors from the endosome to the Golgi apparatus [25]. Interaction of membrane-associated retromer with the cytosolic tail of its cargo (*i.e.*, sorting receptors) triggers transport back to the Golgi apparatus. In yeast and mammalian cells, retromer function also requires a heterodimer of sorting nexins, which are rigid, curved BAR (Bin-Amphiphysin-Rvs) domain proteins capable of deforming membranes and stabilizing endosomal tubules [28]. However, sorting nexins are not universally conserved in eukaryotes and appear to be absent from *P. falciparum* (Results and [29]).

We localized the retromer cargo-selective complex and PfRab7 in asexual blood-stage *P. falciparum* to a putative endosomal compartment. The spatial relationship of the endosome to other subcellular compartments in the parasite was characterized. We describe attempts to perturb protein traffic through the endosome by expressing PfRab7 dominant negative and constitutively active mutants. The effect of blocking COPI-dependent vesicular traffic on endosomal structure was determined by treating parasites with brefeldin A. To gain insight into a possible role for retromer in recycling protein sorting receptors, we characterized the subcellular distribution of the sole *P. falciparum* homolog of the Vps10/sortilin family of protein sorting receptors. Together, these studies define a new compartment in the *P. falciparum* secretory system with a possible role in protein sorting and organelle biogenesis.

## Results

### The *P. falciparum* genome encodes the three retromer cargo-selective subunits

The genome of *P. falciparum* clone 3D7 encodes a single homolog of each retromer cargo-selective subunit: PfVps26 (GeneID PF3D7_1250300), PfVps29 (GeneID PF3D7_1406700), and PfVps35 (GeneID PF3D7_1110500). PfVps26, PfVps29 and PfVps35 share 53, 47 and 30% identity at non-gap positions with the human orthologs Vps26 (isoform A or B), Vps29 and Vps35, respectively (Fig. S1). Residues that contribute to interactions between cargo-selective subunits in human retromer are generally well conserved in the *P. falciparum* sequences (Fig. S1). Transcriptomic data indicate that expression of all three retromer cargo-selective subunits peaks between early trophozoite and late schizont stages [30]–[32].

In addition to the cargo-selective complex, a second component of the retromer complex in mammals and yeast is a PX-BAR sorting nexin dimer, which binds to PI3P through the Phox (PX) domains and induces or stabilizes membrane curvature with the BAR domains [22], [33]–[35]. We attempted to identify parasite sorting nexins using PSI-BLAST searches with human sorting nexin 1 and *S. cerevisiae* Vps5p as queries but were unable to find any *P. falciparum* homologs. When we restricted the search to the PX domains, we found two PX domain-containing proteins in the *P. falciparum* genome but neither appeared to be followed by a BAR domain (data not shown). Our results are consistent with a previous report that sorting nexins are absent from *P. falciparum* and other pathogenic protozoa [29].

### *P. falciparum* retromer labels a putative endosome in asexual blood-stage parasites

To determine whether retromer is associated with an endosome-like compartment in blood-stage *P. falciparum*, we attempted to independently modify the endogenous PfVps26, PfVps29 and PfVps35 loci to encode C-terminal fusions to the enhanced yellow fluorescent protein (YFP) variant “Citrine” [36] or the 9-residue hemagglutinin (HA) tag. Parasite lines expressing PfVps29-YFP, PfVps35-YFP, and PfVps35-HA were obtained (Fig. S2); however, we were unable to introduce a tag (either YFP or HA) at the C-terminus of PfVps26.

**Figure 1:**
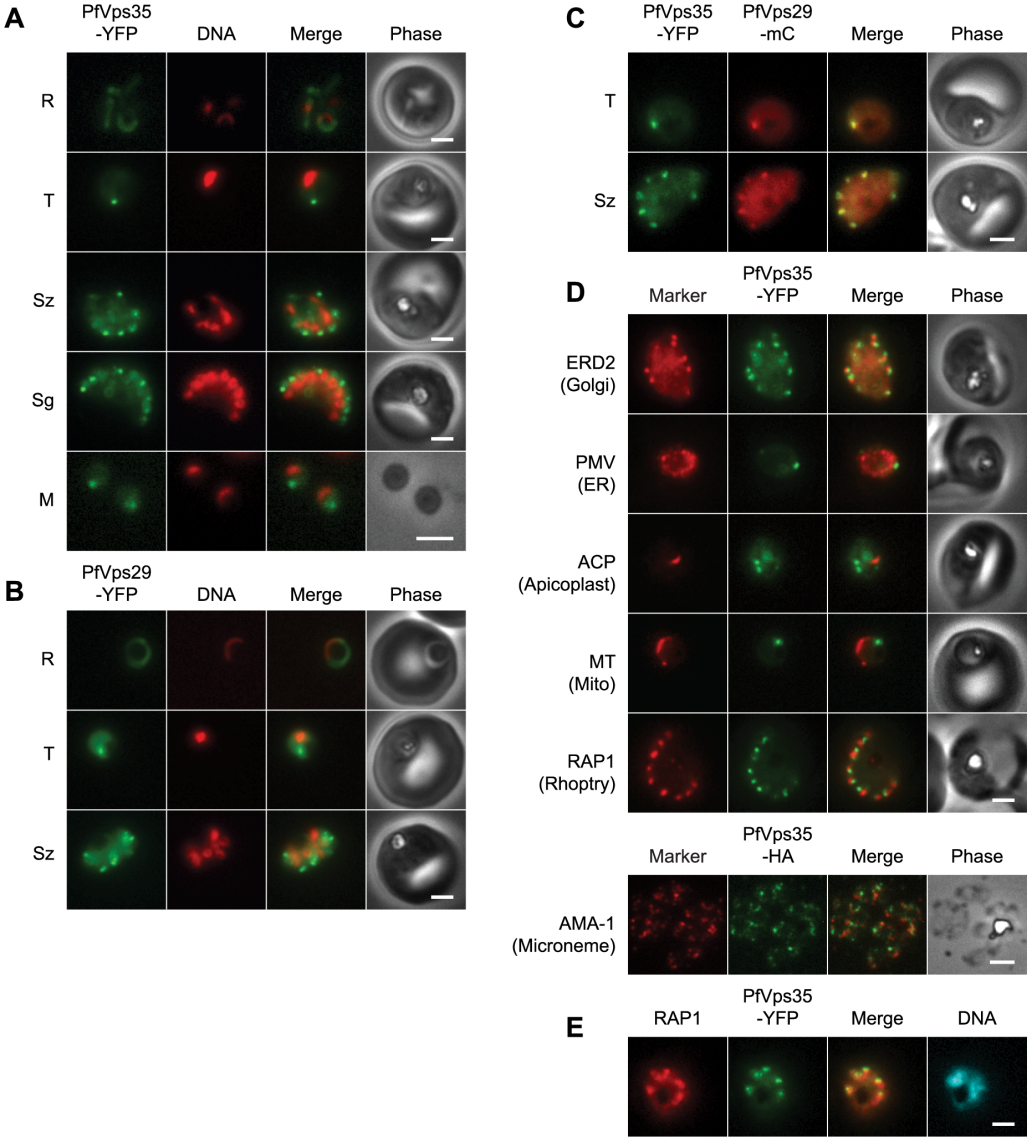
The retromer cargo-selective complex localizes to a novel, heritable subcellular compartment. (A) Wide-field epifluorescence images of live parasites expressing PfVps35-YFP. Parasites are shown at ring (R), trophozoite (T), schizont (Sz), segmenter (Sg), and extracellular merozoite (M) stages. Hoechst 33342 fluorescence (DNA) is pseudocolored red. (B) Images of live parasites expressing PfVps29-YFP. (C) Images of live parasites co-expressing PfVps35-YFP and PfVps29-mCherry in trophozoite (T) and schizont (Sz) stages. mCherry (mC) fluorescence is pseudocolored red. (D) Co-localization of PfVps35-YFP or PfVps35-HA and organellar markers in fixed parasites (except for MitoTracker, which was imaged live). PMV, plasmepsin V; ACP, acyl carrier protein; MT, MitoTracker Red CM-H2Xros; RAP1, rhoptry associated protein 1; AMA1, apical membrane antigen 1. The AMA1 panel shows free merozoites; all others are intraerythrocytic. Organelles labeled by the markers are indicated in parenthesis. Marker-derived fluorescence is pseudocolored red. (E) PfVps35-YFP is adjacent to developing rhoptries in a 2N parasite. Hoechst 33342 fluorescence (DNA) is pseudocolored cyan. In all panels, YFP fluorescence is pseudocolored green. Scale bars, 2 µm.

Both PfVps29-YFP and PfVps35-YFP were expressed throughout the asexual blood stage (Fig. 1). In live parasites expressing PfVps35-YFP, punctate structures were observed in trophozoites and schizonts amid a background of diffuse, presumably cytosolic fluorescence (Fig. 1A). One to two puncta were present in trophozoite stages. As the parasites matured, the puncta became more numerous until in segmenting schizonts there appeared to be one retromer-labeled punctum for each daughter nucleus. Single puncta were present in egressed merozoites, which indicates that the retromer-labeled compartment is inherited. Surprisingly, the PfVps35-YFP-labeled puncta were no longer visible in early ring-stage parasites (Fig. 1A). The distribution of PfVps29-YFP across the asexual cycle was essentially identical to that of PfVps35-YFP (Fig. 1B). To determine whether PfVps35 and PfVps29 are present on the same structures, a transposable PfVps29-mCherry expression cassette was introduced into the genome of the parasite line expressing PfVps35-YFP. The two fluorescent proteins co-localized to the same punctate structures in trophozoites and schizonts (Fig. 1C).

To establish the position of the retromer-labeled compartment in relation to other known organelles, PfVps35-YFP and PfVps35-HA were co-localized with organellar markers (Fig. 1D). Localization of the Golgi apparatus with antibodies against ERD2 revealed that this organelle and the PfVps35-YFP-labeled compartment are distinct structures with a close spatial relationship (Fig. 1D). A similar result was obtained with the Golgi-specific protein PfRab6 as described below. Thus, the PfVps35-YFP-labeled compartment appears to be adjacent to yet distinct from the *P. falciparum* Golgi apparatus. There was no apparent overlap of PfVps35-YFP with markers for the endoplasmic reticulum, apicoplast, mitochondrion or micronemes (Fig. 1D).

In the schizont stage, the rhoptries and micronemes are generated *de novo*. Interestingly, the PfVps35-YFP-labeled compartment was consistently found in close proximity to the rhoptry (Fig. 1D and E). This spatial relationship was observed at the earliest time points at which the rhoptry marker, rhoptry-associated protein 1 (RAP1), was visible (*i.e*., as nuclear division commenced) and before the organelles segregated into individual merozoites (Fig. 1E). Taken together, the localization studies reveal that *P. falciparum* retromer is located on a subcellular compartment that is distinct from known components of the *P. falciparum* endomembrane system. On the basis of its association with the retromer cargo-selective complex, we term this compartment the “*P. falciparum* endosome”. Close spatial proximity to the Golgi apparatus and to developing rhoptries raises the intriguing possibility that the endosome plays a role in protein trafficking to this apical organelle.

### Attempt to perturb retromer function

To establish the importance of retromer in asexual blood stages and gain insight into the role of the endosome in protein trafficking, we attempted to disrupt the coding sequence of PfVps35 using double-crossover homologous recombination coupled with positive and negative selection [37]. This strategy would replace the sequence coding for residues 244 to 817 with a drug selection cassette (Fig. S2). PfVps35 was chosen for disruption because it is the central component of the retromer cargo-selective complex and interacts with both PfVps29 and PfVps26 [38]. Thus, the absence of full-length PfVps35 would preclude the formation of a functional retromer complex. Three independent sets of transfected parasites were subjected to multiple rounds of positive-selection drug cycling followed by negative selection, with the result that the PfVps35 sequence remained intact (Fig. S2). The inability to disrupt the PfVps35 coding sequence suggests that the retromer cargo-selective complex could be important for efficient asexual blood-stage growth.

### The small GTPase PfRab7 co-localizes with retromer to the endosome

In light of our inability to perturb retromer function by gene disruption, we sought an alternate means of dysregulating protein traffic through the *P. falciparum* endosome. Dominant negative and constitutively active mutants of Rab family GTPases are useful tools for interrogating trafficking pathways in yeast and mammalian systems. Rab7 is a well-established marker of the late endosome in mammalian cells and is required for recruitment of the retromer cargo-selective complex to the endosomal membrane [26], [27], [39], [40]. The *P. falciparum* genome encodes one Rab7 homolog, designated as PfRab7 (GeneID PF3D7_0903200) for which transcription is maximal in trophozoite and early schizont stages [41]–[43]. Thus, we investigated the subcellular distribution of PfRab7 and explored whether the expression of PfRab7 mutants could provide insight into the role of the putative *P. falciparum* endosome.

**Figure 2:**
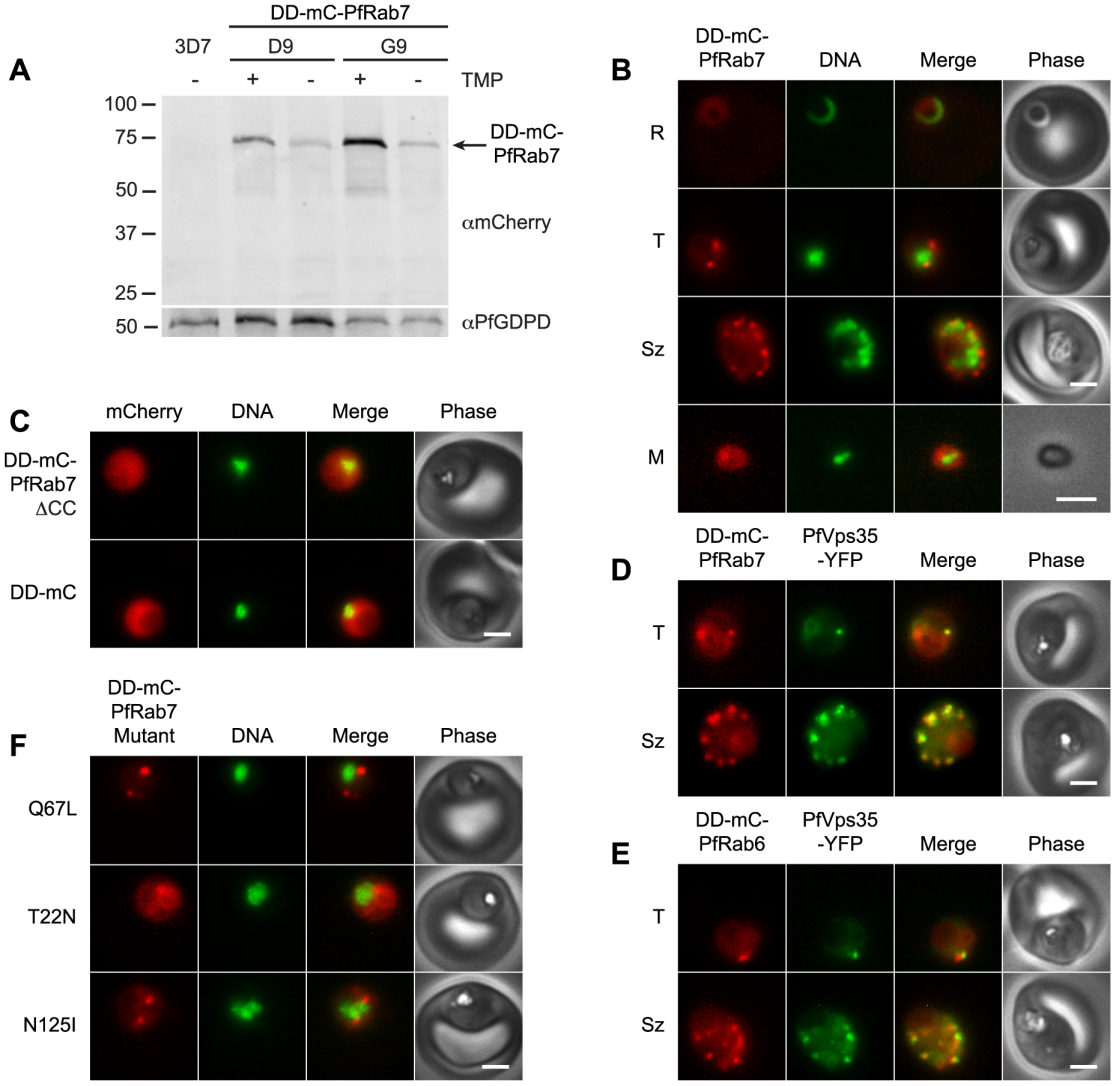
PfRab7 localizes to the same compartment as the retromer cargo-selective complex. (A) Effect of the DD-stabilizing agent trimethroprim (TMP) on the steady-state levels of DD-mCherry-PfRab7 in clonal parasite lines D9 (one expression cassette per genome) and G9 (two expression cassettes per genome). 3D7 is the untransfected parental parasite line. Upper: Anti-mCherry immunoblot. Lower: The membrane was reprobed with anti-glycerophosphoester phosphodiesterase (PfGDPD) antibodies for a loading control. The sizes of protein markers in kDa are indicated at left. (B) Images of DD-mCherry-PfRab7 in live clone G9 parasites. Stages shown are ring (R), trophozoite (T), schizont (Sz), and extracellular merozoite (M). (C) Live parasites expressing DD-mCherry-PfRab7ΔCC and DD-mCherry. (D) Live parasites co-expressing PfVps35-YFP and DD-mCherry-PfRab7. (E) Live parasites co-expressing PfVps35-YFP and DD-mCherry-PfRab6. (F) Live parasites expressing DD-mCherry-PfRab7 mutants. T22N and N125I are predicted to be “GDP-locked” and Q67L is predicted to be “GTP-locked”. 5′ UTR sequences driving expression are *pfrab7* for N125I and Q67L and *pfapp* (aminopeptidase P) for T22N. In panels B, C and F, Hoechst 33342 fluorescence (DNA) is pseudocolored green and in panels D and E YFP fluorescence is pseudocolored green. In panels B–F, parasites were cultured in the presence of 10 µM trimethoprim. mCherry (mC) fluorescence is pseudocolored red. Scale bars, 2 µm.

We first determined whether steady-state levels of mCherry-PfRab7 expressed from a transposable cassette in *P. falciparum* could be regulated by N-terminal fusion to the *E. coli* DHFR destabilization domain (DD). Regulated expression would be necessary to mitigate any deleterious effects of PfRab7 mutants during parasite transfection and selection. A transposable DD-mCherry-PfRab7 expression cassette under the control of the PfRab7 promoter was generated and transfected into wild-type parasites. Two clonal parasite lines were obtained (Fig. S2). One line (D9) contained one copy of the transposon expression cassette, whereas the other line (G9) possessed two copies. In both clones, steady-state levels of the DD-mCherry-PfRab7 fusion were ∼5-fold higher in the presence of the DD-stabilizing ligand trimethoprim (Fig. 2A). Full-length DD-mCherry-PfRab7 (predicted size 69 kDa) was the major species detected with anti-mCherry antibodies in both parasite lines.

DD-mCherry-PfRab7 exhibited a dual cytosolic-punctate distribution in the presence of trimethoprim (Fig. 2B). In trophozoites one to two punctate structures were observed. These puncta multiplied as parasites matured and were segregated to daughter merozoites in segmented schizonts. In free merozoites, concentrated foci of DD-mCherry-PfRab7 could be observed; however, as in the case of PfVps35-YFP, no puncta were found in ring-stage parasites. The cytosolic fluorescence likely reflects the distribution of inactive, GDP-bound PfRab7 whereas the punctate distribution is presumably attributable to the membrane-bound PfRab7-GTP complex. To confirm that the puncta were labeled with prenylated, membrane-bound DD-mCherry-PfRab7, we expressed a truncated variant of PfRab7 lacking the C-terminal two-amino acid prenylation motif required for membrane insertion (DD-mCherry-PfRab7ΔCC; Fig. S1). In the presence of trimethoprim, DD-mCherry-PfRab7ΔCC fluorescence was distributed throughout the parasite cytosol and no puncta were observed (Fig. 2C). This distribution was indistinguishable from that of DD-mCherry expressed alone (Fig. 2C). We conclude that the puncta observed with DD-mCherry-PfRab7 reflect the distribution of the active, membrane-bound PfRab7-GTP complex.

To determine whether PfRab7 was present on the same subcellular structures as the retromer cargo-selective complex, the DD-mCherry-PfRab7 transposable expression cassette was introduced into the genome of the parasite line expressing PfVps35-YFP. Substantial overlap of mCherry and YFP fluorescence was observed in trophozoite- and schizont-stage parasites (Fig. 2D). To confirm that this co-localization was specific for PfRab7, we also generated parasites co-expressing the Golgi protein PfRab6 (expressed as a DD-mCherry-PfRab6 fusion from the *pfrab6* promoter) and PfVps35-YFP. As described above for the Golgi marker ERD2, PfVps35-YFP-labeled puncta were located adjacent to DD-mCherry-PfRab6-labeled Golgi compartments but the two proteins did not co-localize (Fig. 2E). Together, these results indicate that PfRab7 and retromer are both found on the *P. falciparum* endosome and confirm the close spatial proximity of this compartment to the parasite's Golgi apparatus.

### Expression of PfRab7 dominant negative and constitutively active mutants in *P. falciparum*

Dominant negative mutants of Rab7 are impaired for GTP binding [44], [45] and exert their effects by sequestering Rab effector proteins. In contrast, constitutively active mutants exhibit greatly reduced intrinsic GTPase activity [44], [46] and persist in an activated, membrane- and GTP-bound state. Both types of mutants can potentially disrupt specific vesicle trafficking pathways and provide insight into the role of the endogenous Rab protein. By reference to the literature on Rab7 mutants cited above and sequence alignments, we identified T22N and N125I as potential dominant negative mutants and Q67L as a potential constitutively active mutant of PfRab7 (Fig. S1). We generated transposable cassettes to express DD-mCherry fusions of the three PfRab7 mutants and introduced them into parasites. Expression of the fusions was driven by either the native *pfrab7* promoter or, to potentially increase the steady-state levels, by the stronger *P. falciparum* aminopeptidase P promoter (S. Dalal and M. Klemba, unpublished data). Parasite lines conditionally expressing all three PfRab7 mutants were obtained with both promoters (Fig. 2F and data not shown).

When expressed in mammalian cells, Rab7 dominant negative mutants (T22N and N125I) are found in the cytosol and have a profound effect on late endosome-to-lysosome trafficking and lysosome morphology [44], [47], [48]. Lysosome-bound vesicular transport requires multiple effectors that interact with active GTP-bound Rab7. For example, dominant negative Rab7 mutants prevent complex formation with dynein-dynactin motors that direct minus-end trafficking on microtubules towards the lysosome [49], [50]. As a result, the lysosome becomes dispersed into small vesicles that have reduced acidity and are not accessible to endocytic traffic [44]. When expressed in *P. falciparum* from either the *pfrab7* or the *pfapp* promoter and stabilized with trimethoprim, the T22N mutant had the expected cytosolic distribution (Fig. 2F), which suggests that it exists to a greater extent in the GDP-bound state than wild-type PfRab7. Surprisingly, the N125I mutant was present in a punctate distribution that resembled that of wild-type PfRab7, an observation that suggests that this mutant persisted in a GTP-bound state (Fig. 2F). Neither PfRab7 mutant inhibited parasite growth or altered the morphology of the lysosome-like food vacuole as assessed by phase contrast microscopy (data not shown).

The constitutively active Q67L mutant of human Rab7 associates with the lysosomal membrane and increases the rate of endosome-to-lysosome fusion, resulting in an enlarged perinuclear acidic organelle labeled with both lysosome and late endosomal proteins [44], [51]. In this study, DD-mCherry-PfRab7Q67L exhibited a very similar distribution to that of wild-type PfRab7 (Fig. 2F). Microscopic inspection of live parasites indicated that food vacuole morphology was normal. Live-cell fluorescence and immunofluorescence experiments revealed that DD-mCherry-PfRab7Q67L had not redistributed to the Golgi apparatus, rhoptries or food vacuole (data not shown). To all appearances, PfRab7Q67L behaved indistinguishably from wild-type PfRab7.

### PfRab6 but not PfRab7 or retromer redistributes to the ER upon brefeldin A treatment

The fungal metabolite brefeldin A is widely used to interrogate endomembrane trafficking pathways. Addition of brefeldin A to mammalian cells rapidly induces the reversible collapse of the Golgi apparatus into the ER [52], [53], endosome tubulation [54]–[56] and recycling endosome fusion to the *trans*-Golgi network [57], [58]. Early-to-late endosome maturation is also disrupted [54], [59], [60]. The mechanism of action involves the inhibition of three guanine nucleotide exchange factors (GEFs) that act on small GTPases of the ADP-ribosylation factor (Arf) family at the *cis*- or *trans*-Golgi cisternae and *trans*-Golgi network [61]–[64]. Brefeldin A also stabilizes the inactive GDP-Arf GTPase and Arf-GEF complexes [62], [65], which can no longer properly recruit COP1 or AP-1/clathrin coat proteins [66].

**Figure 3:**
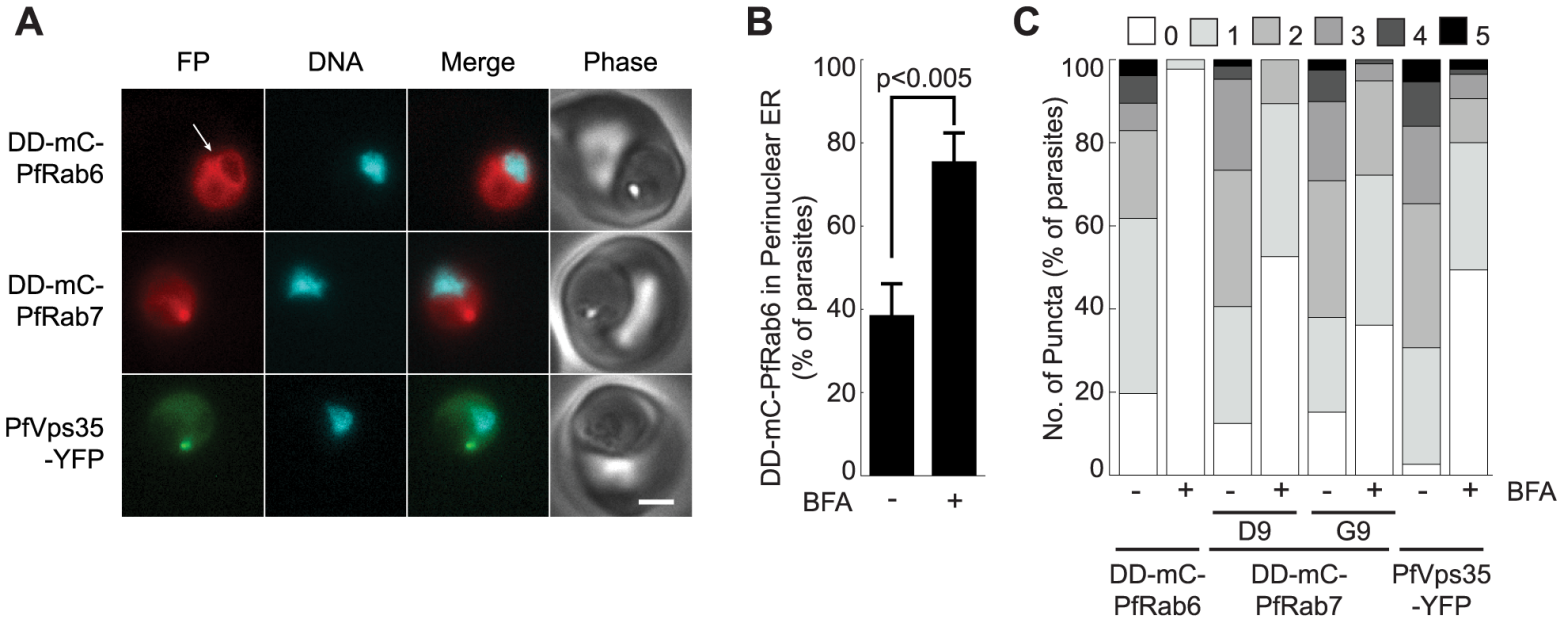
PfRab6 but not PfRab7 or retromer rapidly redistributes to the ER upon brefeldin A treatment. (A) Distributions of DD-mCherry-PfRab6, DD-mCherry-PfRab7 and PfVps35-YFP in live parasites after one hour in the presence of 5 µg/mL brefeldin A. Redistribution of DD-mCherry-PfRab6 to the perinuclear ER is indicated with an arrow (top panel). FP, fluorescent protein. mCherry fluorescence is pseudocolored red, YFP fluorescence is pseudocolored green and Hoechst 33342 fluorescence is pseudocolored cyan. Scale bar, 2 µm. (B) Effect of brefeldin A (5 µg/mL, 1 hour) on the percentage of parasites exhibiting ER-associated DD-mCherry-PfRab6 fluorescence. Results are an average of three experiments, n = 75 to 85 parasites per condition. The *p*-value was determined using a two-tailed Student's t-test. (C) Effect of Brefeldin A (5 µg/mL, 1 hour) on the number of puncta labeled with DD-mCherry-PfRab6, DD-mCherry-PfRab7 (clones D9 and G9) or PfVps35-YFP. Results are averages of three experiments, n = 50 to 70 parasites per condition.

We compared the effects of brefeldin A on the *P. falciparum* Golgi apparatus and endosome by treating parasites lines expressing DD-mCherry-PfRab6 and PfVps35-YFP together or DD-mCherry-PfRab7 alone with 5 µg/mL brefeldin A for one hour. As expected, the Golgi marker DD-mCherry-PfRab6 redistributed to the perinuclear ER (Fig. 3A). After the one hour brefeldin A treatment, fewer than 3% of parasites contained a DD-mCherry-PfRab6-labeled punctum, compared to 80% in the untreated control (Fig. 3C). In addition, the number of parasites exhibiting DD-mCherry-PfRab6 fluorescence at the perinuclear ER doubled (Fig. 2B). The effect of brefeldin A on the endosome was much less pronounced. We observed a reduction in the number of PfVps35-YFP- and DD-mCherry-PfRab7-labeled puncta, but such structures persisted in about half of the parasites (Fig. 3C). Association with the perinuclear ER was not observed for either protein (Fig. 3A). The contrasting responses of the Golgi apparatus and endosome to brefeldin A treatment reveal differences in the dynamics of vesicular traffic to and from these compartments.

### PfSortilin, a putative retromer cargo, is located in the Golgi apparatus

**Figure 4:**
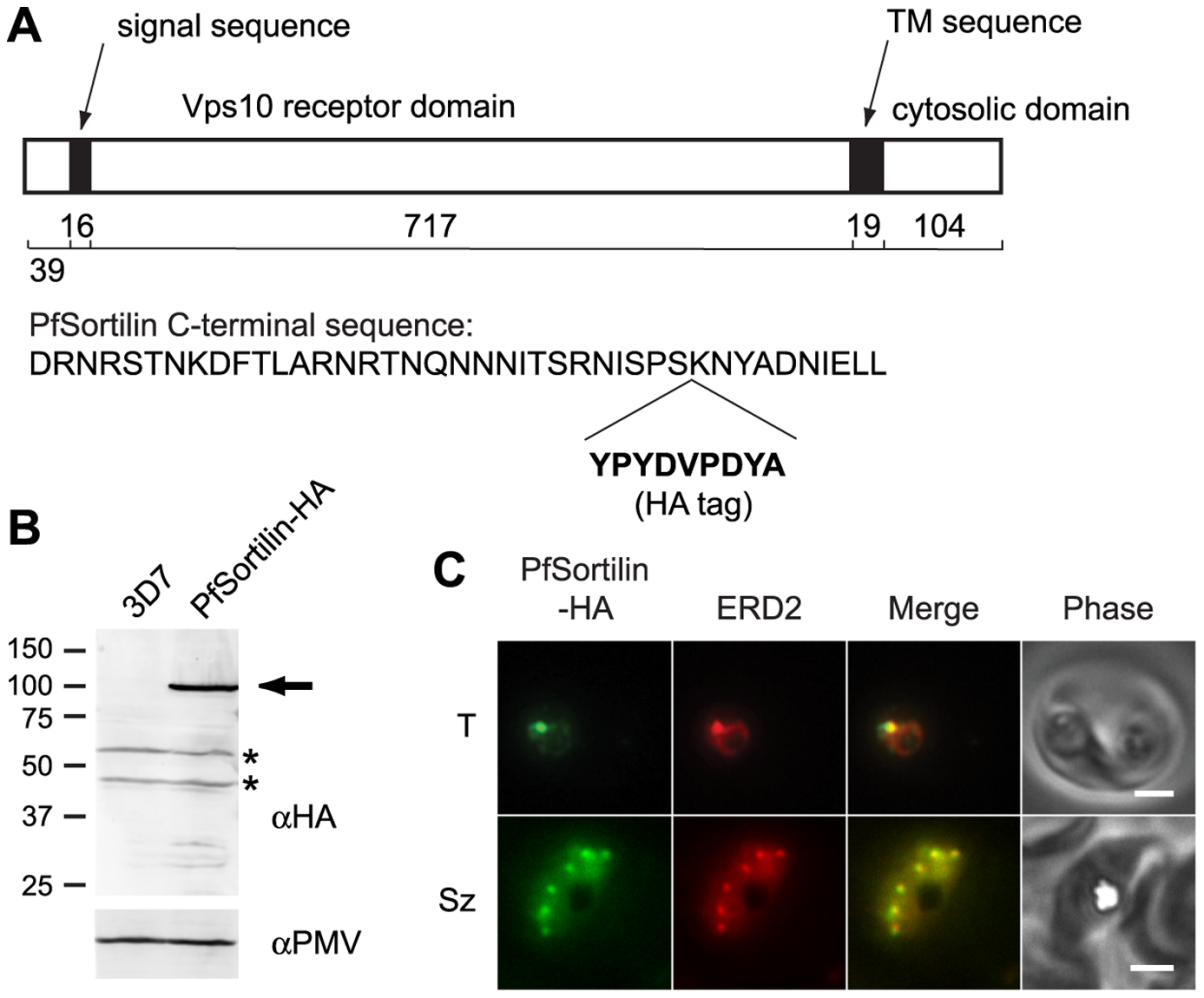
The putative protein sorting receptor PfSortilin localizes to the *P. falciparum* Golgi apparatus. (A) Schematic diagram of the domain organization of PfSortilin with the number of amino acids in each domain indicated below. At bottom is the C-terminal sequence of PfSortilin with the site of incorporation of the HA tag indicated. TM, transmembrane. (B) Anti-HA immunoblot of the membrane fraction of parasites expressing PfSortilin-HA (clone C9) and of the parental 3D7 parasite line. PfSortilin-HA is indicated with an arrow. Two cross-reacting species are present in both lanes (asterisks). The membrane was reprobed with anti-plasmepsin V (PMV) antibodies for a loading control. The sizes of protein markers in kDa are indicated at left. (C) Co-localization of PfSortilin-HA and ERD2 in aldehyde-fixed clone C9 parasites. T, trophozoite; Sz, schizont. Scale bar, 2 µm.

A major role for the cargo-selective retromer complex in yeast and mammalian cells involves the recycling of protein sorting receptors such as the mannose-6-phosphate receptor (MPR) and the Vps10p-domain-containing sorting receptors known as sortilins from the endosome back to the Golgi apparatus [67]. To assess whether this might be a plausible role for retromer in *P. falciparum*, we searched the parasite genome for MPR and sortilin homologs. We found one sortilin homolog, referred to here as PfSortilin (GeneID: PF3D7_1451800, annotated as “sortilin, putative”) but could not identify any MPR homologs. The PfSortilin open reading frame codes for a 102 kDa protein with a putative signal peptide, a Vps10 domain, a putative single-pass transmembrane sequence, and a C-terminal tail (Fig. 4A). Transcriptomic data indicate that PfSortilin is maximally expressed from about 20 to 40 hours post-invasion (*i.e*., during the trophozoite and schizont stages) [30]–[32].

To localize PfSortilin in asexual blood-stage parasites, we attempted to modify the endogenous locus by single-crossover recombination to encode a C-terminal fusion to YFP. We were unable to obtain parasites with the desired integration and considered the possibility that a C-terminal YFP tag interfered with the normal function of PfSortilin. We therefore attempted to introduce a hemagglutinin epitope tag at an internal position 10 amino acids away from the C-terminus (Fig. 4A), which is a region of low conservation in Apicomplexan sortilin homologs, and obtained the desired recombination event (Fig. S3).

A protein of the size expected for PfSortilin-HA was detected in the membrane fraction of transgenic but not parental parasites upon immunoblotting with an anti-HA antibody (Fig. 4B). In trophozoite- and schizont-stage parasites, PfSortilin-HA localized to puncta that were also recognized by antibodies to the Golgi marker ERD2 (Fig. 4C). In ring-stage parasites, PfSortilin-HA could not be detected above non-specific background, even though the Golgi apparatus was present as determined by ERD2 immunolocalization (data not shown). No Golgi labeling with anti-HA antibodies was observed in wild-type parasites. Thus, at steady state the putative protein sorting receptor PfSortilin is present mainly in the parasite's Golgi apparatus where it presumably interacts with and sorts proteins transiting the endomembrane system.

## Discussion

We have shown that *P. falciparum* homologs of the retromer cargo-selective complex and of the small GTPase Rab7 localize to an intracellular compartment that, to our knowledge, has not been previously described. This compartment appears to be a component of the parasite's endomembrane system and we propose to call it the “*P. falciparum* endosome”. We note that the assignment of the term “endosome” to this structure is based solely on the localization of conserved endosomal proteins and that there is as of yet no functional evidence that this structure has properties associated with endosomes in mammalian cells. With this caveat in mind, we will refer to the plasmodial structure as an “endosome” at this time for the sake of clarity. In terms of its Rab identity, it resembles the mammalian late (Rab7) endosome [22]. It is not clear whether the early endosome, which in mammalian cells is decorated with the lipid PI3P and Rab5, exists as a discrete compartment in *P. falciparum*. PI3P has been localized to the food vacuole, the apicoplast and the luminal face of the ER [68], [69], none of which appears to represent a canonical early endosome.

In contrast to the abundance of late endosomes present in mammalian cells (e.g., [26]), we observe only one or two endosomes in trophozoite stage parasites. This compartment multiplies during schizogony and is inherited by daughter merozoites. In this regard, it behaves more like a Golgi cisterna than like the mammalian late endosome, which terminates its independent existence by fusing with the lysosome. Curiously, the punctate concentrations of the retromer cargo-selective complex and PfRab7 that define the parasite endosome are not visible during the ring stage of the asexual cycle. This may reflect the dissociation of retromer and PfRab7 from the endosome membrane during this stage; alternately, the endosome may undergo a cycle of destruction and regeneration early in the asexual cycle.

Our observations suggest that the *P. falciparum* endosome plays a role in protein trafficking. This compartment resides in close proximity to the Golgi apparatus (as defined by PfRab6 or ERD2) during the trophozoite and schizont stages of the asexual intraerythrocytic cycle. The Golgi apparatus, in turn, has been shown to be close to transitional ER sites where cargo is organized for delivery to the Golgi [21]. The close spatial organization of the transitional ER, Golgi apparatus and endosome could serve to limit the distance that coated vesicles (COPII-coated from the ER, COPI- or clathrin-coated from the Golgi apparatus) must travel between compartments, thus increasing the efficiency of vesicular traffic. Our observations also suggest that there is a tethering mechanism that holds the endosome close to the Golgi apparatus. In mammalian cells, there is evidence that Golgi reassembly stacking proteins (GRASPs) are in part responsible for interactions between Golgi cisternae (*i.e.*, stacking) [70]. We speculate that the *P. falciparum* GRASP homolog, which exists in two isoforms [71], may contribute to tethering the Golgi apparatus and the endosome. Whether or not the two compartments are tethered, the dynamics of vesicular traffic into and out of these compartments are clearly distinct. This was illustrated by treatment of *P. falciparum* with brefeldin A, which caused redistribution of PfRab6 but not PfRab7 or PfVps35 to the endoplasmic reticulum.

The idea that vesicular traffic occurs between the Golgi apparatus and the endosome is supported by our identification of a putative protein sorting pathway between these compartments. We have shown that a *P. falciparum* homolog of the sortilin/Vps10 class of sorting receptor localizes to the Golgi apparatus. In *S. cerevisiae* and mammalian cells, sortilin cycles between the *trans*-Golgi network and the endosome, with the retrograde pathway dependent on the interaction of the cytoplasmic tail of sortilin with the retromer complex [72]. Given the presence of the retromer cargo-selective complex on the *P. falciparum* endosome, we speculate that PfSortilin sorts specific cargo from the Golgi to the endosome, with subsequent recycling to the Golgi mediated by the plasmodial retromer cargo-selective complex. Yeast and mammalian retromer interact with a short hydrophobic motif in the cytosolic tail of the respective sortilin homologs (FYVFSN in yeast, FLV in mammals) [25]. Although neither of these sequences is found in the cytosolic tail of PfSortilin, there are similar hydrophobic sequences (e.g. LFYNY) that could fulfill the same role.

The question then arises as to what biosynthetic cargo might pass through the endosome, and to where. In the course of our immunofluorescence studies, we noticed that the rhoptry bulb protein RAP1 consistently localized to a structure close to the endosome. This proximity was observed in maturing parasites that had not yet begun to segregate organelles into daughter merozoites. Thus, the close proximity of the endosome and the nascent rhoptries did not appear to be merely a consequence of being restricted to a small space (*i.e.*, at the apical end of the merozoite). Based on these observations we suggest a role for the endosome in rhoptry biogenesis. One intriguing possibility is that rhoptry proteins are transported to the endosome by PfSortilin and from the endosome proceed by vesicular traffic to the rhoptry. This idea is supported by the recent finding that the *Toxoplasma gondii* sortilin homolog (TgSORTLR) sorts rhoptry and microneme proteins in this organism [73]. Although the specific rhoptry cargo molecules that were found to interact with TgSORTLR do not have close homologs in *P. falciparum*, it is likely that a subset of conserved rhoptry proteins interacts with sortilin in both organisms. Unfortunately, none of our several attempts to perturb protein trafficking through the endosome was successful and we do not at this time have direct evidence for the trafficking of rhoptry proteins through the *P. falciparum* endosome.

Although the *P. falciparum* endosome and the mammalian late endosome are both decorated with Rab7, we suspect that their roles in the respective organisms are quite different. The close association of the *P. falciparum* endosome with the Golgi apparatus throughout the cell cycle (excepting the ring stage) suggests that this compartment is dedicated to sorting biosynthetic protein traffic. Also, the *P. falciparum* endosome does not appear to fuse in its entirety with other organelles, as does the mammalian late endosome with lysosomes. The loss of ESCRT I and II complexes in *P. falciparum* [74] suggests that the parasite does not down regulate cell surface proteins through ubiquitin-dependent sequestration into multivesicular bodies in late endosomes. These considerations are consistent with the prevailing view that the malaria parasite possesses a “minimal” or “stripped-down” endomembrane system [8]. Further studies will be required to better elaborate the form and functions of the *P. falciparum* endosomal network.

## Materials and Methods

### Construction of plasmids for *P. falciparum* transfection

Plasmids for the generation of chimeras between the chromosomal coding sequences of PfVps26, PfVps29 or PfVps35 and that of yellow fluorescent protein (YFP) or an HA tag through single-crossover homologous recombination were produced as follows (see also Fig. S2). One kilobase of the 3′ end (excluding the stop codon) of PfVps26, PfVps29 and PfVps35 coding sequences was PCR amplified from *P. falciparum* 3D7 genomic DNA using oligos 428/429, 334/335, and 352/353, respectively. (All oligonucleotides used in this study are listed in Table S1). The PCR product was introduced into the XhoI and AvrII sites of pPM2CIT2 [75] or pDAP-HA [78], yielding pPfVps26-YFP, pPfVps29-YFP, pPfVps35-YFP and pPfVps35-HA. To introduce an HA tag into the PfSortilin locus, a PCR product encompassing bases 2476 to 3106 of the genomic PfSortilin sequence was amplified with oligos 276/283 and introduced into the XhoI/AvrII sites of plasmid pPM2CIT2. A pair of annealed oligonucleotides (286/287) containing the sequence of the HA tag and bases 3107–3139 of PfSortilin was subsequently introduced into the AvrII/NotI sites to generate pSortilin-HA.

A construct for double-crossover recombination at the PfVps35 locus was produced by PCR amplification of 5′ (bases 368–938) and 3′ (bases 2661–3238) fragments of the PfVps35 open reading frame using oligo pairs 344/345 and 346/347, respectively. These PCR products were cloned into the SacII/SpeI and NcoI/AvrII sites, respectively, in the plasmid pCC-1 [76] to produce pPfVps35-DKO (Fig. S2).

Plasmids for transposon-based expression were constructed from pXL-BacII-DHFR [77]. First, a linker (oligos 245/246) was cloned into the XhoI site to introduce unique KasI and XmaI sites while maintaining a unique downstream XhoI site. Next the XhoI-BglII restriction fragment (containing internal AvrII and NotI sites) from pPM2CIT2 [75] was cloned into the same sites in the modified pXL-BacII-DHFR, yielding pSD-DHFR. To generate an expression cassette for PfVps29-mCherry, the complete coding sequence of PfVps29 (without a stop codon) was PCR amplified with oligos 456/457 and introduced into the XhoI/AvrII sites of pSD-DHFR. The sequence for mCherry (with an N-terminal tobacco etch virus protease site but no start codon) was amplified with oligos 460/461 from pRSET_B_-mCherry [78] and cloned into the AvrII/NotI sites. 0.8 kb of the 5′ UTR of PfVps29 were PCR amplified with oligos 458/459 and introduced into the XmaI/XhoI sites to yield pPfVps29-mCherry. N-terminal DHFR destabilization domain-mCherry (DD-mCherry) fusions to PfRab6 and PfRab7 were constructed as follows. The *E. coli* DHFR destabilization domain (DD) coding sequence (omitting the stop codon) was PCR amplified from pBMN-DHFR(DD)-YFP [79] using oligos 470/471 and cloned into XhoI/AvrII sites of pSD-DHFR. The sequence for mCherry (omitting the start and stop codons) was PCR amplified with oligos 484/485 to contain 5′ AvrII and 3′ SacII/NotI sites and was cloned into AvrII/NotI sites. 0.8 kb of the 5′ UTR for PfRab6 and PfRab7 were amplified using oligos 480/481 and 476/477, respectively, and cloned into XmaI/XhoI sites. cDNA sequences for PfRab6 and PfRab7 were obtained by SuperScript II reverse transcriptase/PCR (Invitrogen) using unsynchronized *P. falciparum* 3D7 total RNA and gene-specific primers. PfRab6 and PfRab7 sequences were then amplified using oligo pairs 482/483 and 478/479, respectively, and cloned into SacII/NotI sites to yield pDD-mCherry-PfRab6 and pDD-mCherry-PfRab7. To generate pDD-mCherry-PfRab7ΔCC, the wild-type PfRab7 coding sequence was replaced with a PfRab7 sequence lacking the codons for the two C-terminal cysteine residues that was obtained by PCR amplification of PfRab7 cDNA using oligos 478/598. To obtain plasmid pDD-mCherry, the mCherry sequence (omitting the start codon but containing a stop codon) was PCR amplified with oligos 484/597 and cloned into AvrII/NotI sites of pDD-mCherry-PfRab7. Finally, the hDHFR cassette in selected transposon expression constructs was excised from the BglII/EcoRI sites and replaced with a yDHOD expression cassette amplified from pUF-1 [80] with oligos 501/502 to yield pPfVps29-mCherry-yDHOD, pDD-mCherry-PfRab6-yDHOD and pDD-mCherry-PfRab7-yDHOD.

To generate point mutants of PfRab7, the coding sequence was PCR amplified with oligos 599/600 to contain 5′ BamHI/SacII and 3′ NotI/XhoI sites and cloned into the BamH1/XhoI sites of pSP72 (Promega). Site-directed mutagenesis was performed using the Quikchange II XL kit (Agilent) and oligo pairs 591/592 for PfRab7T22N, 593/594 for PfRab7Q67L and 595/596 for PfRab7N125I. Mutated sequences were excised with SacII and NotI and cloned into the same sites in pDD-mCherry-PfRab7 to yield pDD-mCherry-PfRab7T22N, pDD-mCherry-PfRab7Q67L, and pDD-mCherry-PfRab7N125I. A second set of mutant PfRab7 mutant expression cassettes with transcription driven by the promoter sequence of *P. falciparum* aminopeptidase P (GeneID PF3D7_1454400) was constructed by replacing the PfRab7 5′ UTR with a PCR product (oligos 251/144) containing bases -865 to -39 of the PfAPP 5′ UTR.

All coding sequences that were subjected to PCR amplification were verified by DNA sequencing. Plasmids used in this study are listed in Table S2.

### Parasite culture and transfection

*P. falciparum* 3D7 parasites were cultured in human O^+^ erythrocytes (Interstate Blood Bank; 2% hematocrit) in RPMI 1640 medium (Life Technologies) supplemented with 27 mM sodium bicarbonate, 11 mM, glucose, 0.37 mM hypoxanthine, 10 µg/mL gentamicin, and 5 g/L Albumax I (Invitrogen). Parasites were synchronized by 5% sorbitol treatment [81]. Parasites used for immunoblotting were isolated from intact red blood cells by treatment with 1.5 mg/mL saponin in phosphate buffered saline.

For experiments involving homologous recombination, ring-stage 3D7 parasites were transfected with 100 µg of plasmid DNA by electroporation using low voltage conditions [82]. Transfected parasites were selected with 10 nM WR99210. Drug-resistant parasites were subjected to a drug cycling protocol whereby parasites were cultured without selection for 3 weeks followed by re-selection. To attempt to isolate parasites that had undergone double-crossover homologous recombination at the PfVps35 locus, the negative selection agent 5-fluorocytosine [37] was added to cultures at 300 nM. To introduce plasmids carrying expression cassettes on the *piggyBac* transposon, uninfected erythrocytes were transfected with 100 µg of transposon-containing plasmid and 50 µg of the helper plasmid pHTH [77]. Late-stage parasites were purified using a MACS magnetic column (Miltenyi Biotec) and electroporated erythrocytes were seeded with 2.5–5×10^5^ parasites/mL. Drug selection with WR99210 (2.5 nM) or DSM-1 (1.5 µM) was initiated two days after transfection and drug-resistant parasites appeared after about 18 days. Clonal parasite lines were generated by limiting dilution.

### Southern blot analysis

Genomic DNA was isolated from saponin-treated parasites using the QiaAmp DNA Mini kit (Qiagen) and digested with one or two restriction enzymes as indicated in Fig. S2. DNA fragments were resolved on a 0.6% agarose gel, transferred to positively-charged nylon membrane and hybridized to labeled probes overnight. Probe labeling and detection were carried out using the AlkPhos direct labeling kit (GE Biosciences) or BrightStar Psoralen-Biotin Non-isotopic labeling kit (Ambion) according to the manufacturer's instructions.

### Immunofluorescence assays and live cell imaging

For live cell imaging, parasites in culture were resuspended and placed under a coverslip following a short (<30 minute) incubation with the vital nuclear stain Hoechst 33342 (5 µM). Images were collected on a Zeiss AxioImager equipped with an MRm Axiocam digital camera using a 100×/1.4NA objective lens. For all immunofluorescence assays except those involving AMA1, parasites were fixed and permeabilized as described previously [75] and incubated with organelle-specific antibodies followed by Alexa 488-or 594-conjugated rabbit or mouse secondary antibodies diluted to 2 µg/mL (Invitrogen). The primary antibodies used were: rabbit anti-ERD2 [11], 1∶500; mouse anti-plasmepsin V [83], 1∶10; rabbit anti-acyl carrier protein [84], 1∶5000; mouse anti-rhoptry associated protein 1 (RAP1) monoclonal antibody 7H8/50 [85], 1∶200; and anti-HA monoclonal antibody 16B12 (Covance), 2 µg/mL. For AMA1 and PfVps35-HA co-localization, smears of late-stage parasites were air-dried on glass coverslips and fixed in a 1∶1 methanol-acetone solution at −20°C for 5 minutes. Samples were first incubated with mouse anti-AMA1 antibody 1G6 (1∶400) followed by anti-mouse Alexa 594-conjugated secondary antibody (Invitrogen) and then with Alexa 488-conjugated mouse anti-HA antibody 16B12 (Covance), 10 µg/mL. DNA was stained with 1 µM Hoechst 33342. For colocalization with mitochondria, parasites were incubated in culture medium containing 100 nM MitoTracker Red CM-H2XRos (Invitrogen) for 15 minutes at 37°C. Cells were then washed into MitoTracker-free medium containing 5 µM Hoechst 33342 and were imaged live. For figure preparation, images were converted to TIF files and contrast was adjusted using Adobe Photoshop CS6.

For analysis of the effects of brefeldin A, live parasites expressing DD-mCherry-PfRab7 or co-expressing DD-mCherry-PfRab6 and PfVps35-YFP were cultured in media containing 10 µM trimethoprim and 0.2% DMSO with or without 5 µg/mL brefeldin A for 1 hour. To ensure that parasites of a similar stage of development were compared, only single-parasite infected erythrocytes with one to two nuclei (as determined by Hoechst 33342 fluorescence) and a visible hemozoin crystal (*i.e*., trophozoite stage) were included in the analysis. The numbers of fluorescent puncta and the association of fluorescence with the perinuclear ER were determined by visual inspection of images.

### Immunoblotting

Saponin parasite pellets were lysed by sonication in phosphate buffered saline with protease inhibitors (10 µM E–64, 10 µM pepstatin A and 1 mM 4-(2-aminoethyl)benzenesulfonyl fluoride). Lysates were centrifuged at 16,100× *g* for 10 minutes at 4°C to remove hemozoin. Samples from parasites expressing PfSortilin-HA were further centrifuged at 100,000× *g* for 1 hour at 4°C to isolate the membrane fraction, which was solubilized in reducing, SDS-containing Laemmli buffer. Proteins were resolved on polyacrylamide gels and blotted to nitrocellulose. Antibodies used were anti-RFP (Rockland), 0.3 µg/mL; anti-HA monoclonal antibody 16B12 (Covance), 1 µg/mL; anti-plasmepsin V [83], 1∶400; and affinity purified rat anti-PfGDPD [86], 1∶5000. Signal was detected by chemiluminescence using horseradish peroxidase-conjugated anti-rabbit or anti-mouse secondary antibodies. Blots were developed using ECL Plus (GE Biosciences) and imaged on a Storm 840 imager (GE Biosciences) or on film.

## Supporting Information

Figure S1 **Sequence alignments of** ***Plasmodium falciparum*** **retromer cargo-selective subunits and Rab7 with human and** ***Saccharomyces cerevisiae*** **homologs.** Multiple sequence alignments were generated using the Clustal W algorithm in MegAlign version 10.0.3 (DNAStar) and T-Coffee Expresso [87]–[89]. Alignments were manually edited and shaded in Jalview [90]. Light grey shading indicates identity between two sequences and dark gray shading indicates identity among all three sequences. Residue numbers are indicated at left and right. Pf, *Plasmodium falciparum*; Hs, *Homo sapiens*; Sc, *Saccharomyces cerevisiae*. (A) Vps29 alignment. Residues important for Vps35 binding are indicated with squares. The leucine residue critical for binding of Vps5p (yeast) or TBC1D5 Rab GTPase activating protein (human) is indicated with a star. (B) Vps26 alignment. The interdomain loop is indicated with a black bar and the region that interacts with Vps35 is indicated with a double red line. (C) Vps35 alignment. The conserved Vps26-interacting motif is indicated with a bar. (D) Rab7 alignment. Residues mutated in this study are indicated with a square (T22N), circle (Q67L) and star (N125I). The C-terminal prenylation motif is indicated with a red box.
(PDF)

Figure S2 **Southern blot characterization of transgenic parasite lines.** In each panel, a schematic diagram of the product of recombination or transposition is provided on the left. The predicted sizes of DNA fragments upon digestion with the indicated restriction enzyme are shown. The red bar indicates the position of the probe used for Southern blotting. “X” indicates the expected site(s) of homologous recombination, where applicable. Figures are not drawn to scale. Southern blots of genomic DNA from parental and transfected parasite lines are shown on the right. Each blot was performed on a single membrane. For clarity some lanes are shown as individual strips but all strips originate from the same exposure. Sizes of DNA markers in kilobases are indicated at left. Arrows at right identify the positions of the expected bands. The presence of a “plasmid” band in the transfected parasite lines in A, B and E likely reflects the integration of concatameric episomes. Abbreviations: hDHFR, human dihydrofolate reductase; YFP, yellow fluorescent protein; CD, cytosine deaminase; DD, *E. coli* DHFR destabilization domain; HA, hemagglutinin epitope tag. (A) Parasite line expressing PfVps29-YFP generated by single-crossover recombination with pPfVps29-YFP. (B) Parasite line expressing PfVps35-YFP generated by single-crossover recombination with pPfVps35-YFP. (C) Parasite line transfected with pPfVps35-DKO and selected for double-crossover disruption of the PfVps35 coding sequence. (D) Two clonal parasite lines (D9 and G9) obtained by transfection with a plasmid (pDD-mCherry-PfRab7) carrying a transposable DD-mCherry-PfRab7 expression cassette. (E) Parasite line expressing PfVps35-HA following single-crossover recombination with pPfVps35-HA.
(PDF)

Figure S3 **Generation of a parasite line expressing PfSortilin-HA.** (A) Schematic diagram of the single-crossover strategy for modifying the PfSortilin chromosomal locus to incorporate an internal hemagglutinin (HA) tag (indicated by the yellow box). Primers used for PCR analysis are indicated with red arrows and predicted sizes of amplified regions are shown. The figure is not drawn to scale. hDHFR, human dihydrofolate reductase. (B) PCR analysis of genomic DNA obtained from clonal parasite line C9 expressing PfSortilin-HA and from the parental 3D7 line using primers indicated in (A). Sizes of DNA markers are shown at left. The presence of a band with primers 279/HSPR1 (lower panel) is consistent with the expected integration event in clone C9. The primer pair 279/282 serves as a PCR control.
(PDF)

Table S1 **Sequences of oligonucleotides used in this study.**
(PDF)

Table S2 **Plasmids generated in this study.**
(PDF)
