# Supplementary material for: Evidence for a Golgi-to-Endosome Protein Sorting Pathway in Plasmodium falciparum
Source: PLoS One. 2014 Feb 25;9(2):e89771. doi: 10.1371/journal.pone.0089771 (PMC3934947; doi:10.1371/journal.pone.0089771)
Supplement: Figure S1 — Sequence alignments of Plasmodium falciparum retromer cargo-selective subunits and Rab7 with human and Saccharomyces cerevisiae homologs. Multiple sequence alignments were generated using the Clustal W algorithm in MegAlign version 10.0.3 (DNAStar) and T-Coffee Expresso [87]–[89]. Alignments were manually edited and shaded in Jalview [90]. Light grey shading indicates identity between two sequences and dark gray shading indicates identity among all three sequences. Residue numbers are indicated at left and right. Pf, Plasmodium falciparum; Hs, Homo sapiens; Sc, Saccharomyces cerevisiae. (A) Vps29 alignment. Residues important for Vps35 binding are indicated with squares. The leucine residue critical for binding of Vps5p (yeast) or TBC1D5 Rab GTPase activating protein (human) is indicated with a star. (B) Vps26 alignment. The interdomain loop is indicated with a black bar and the region that interacts with Vps35 is indicated with a double red line. (C) Vps35 alignment. The conserved Vps26-interacting motif is indicated with a bar. (D) Rab7 alignment. Residues mutated in this study are indicated with a square (T22N), circle (Q67L) and star (N125I). The C-terminal prenylation motif is indicated with a red box. (PDF) [file pone.0089771.s001.pdf]

Figure S1. Sequence alignments of *Plasmodium falciparum* retromer cargo-selective subunits and Rab7 with human and *Saccharomyces cerevisiae* homologs.

A. Vps29

```
PfVps29      1 MSGKLEDIGELVLLIGDFHSPIRNLGLPDCFKELL-KTDKIKHVLCTGN-VG 50
HsVps29-1    1 ----------MLVLVLGDLHIPHRCNSLPAKFKKLL-VPGKIQHILCTGN-LC 41
ScVps29      1 ----------MLLLALSDAHIPDRATDLPVKFKKLLSVPDKISQVALLGNSTK 43

PfVps29     51 CNENLELLKNIADSVHITKGDMDDN-----------------FDFPEDITLC 85
HsVps29-1   42 TKESYDYLKTLAGDVHIVRGDFDEN-----------------LNYPEQKVVT 76
ScVps29     44 SYDFLKFVNQISNNITIVRGEFDNGHLPSTKKDKASDNSRPMEEIPMNSIIR 95

PfVps29     86 IGDFKISLIHGHQIIPWGDMNALLQWQKKYDSDIIISGHTHKNSIVQYEGKY 137
HsVps29-1   77 VGQFKIGLIHGHQVIPWGDMASLALLQRQFDVDILISGHTHKFEAFEHENKF 128
ScVps29     96 QGALKIGCCSGYTVVPKNDPLSLLALARQLDVDILLWGGTHNVEAYTLEGKF 147

PfVps29    138 FINPGSVTGAFQ---------------------------------------- 149
HsVps29-1  129 YINPGSATGAYN---------------------------------------- 140
ScVps29    148 FVNPGSCTGAFNTDWPIVFDVEDSDEAVTSEVDKPTKENQSEDDDAKGGSTG 199

PfVps29    150 -----------------------------------------PWLSEPTPTFI 160
HsVps29-1  141 -----------------------------------------ALETNIIPSFV 151
ScVps29    200 KEQPGSYTPKEGTAGERENENESNVKPENQFKEDEVDMSDSDINGSNSPSFC 251

PfVps29    161 LMAVAKSNIVLYVYEEKNGKTNVEMSELHKSTVI                   194
HsVps29-1  152 LMDIQASTVVTYVYQLIGDDVKVERIEYKKP                      182
ScVps29    252 LLDIQGNTCTLYIYLYVNGEVKVDKVVYEKE                      282
```

B. Vps26

```
PfVps26      1 --MLSTIFGSVCSIDLKIDADDNKKFAFLRK-------DKKGEKCPIFSDGE 43
HsVps26a     1 MSFLGGFFGPICEIDIVLNDGETRKMAEMKTE------DGKVEKHYLFYDGE 46
ScVps26      1 ---MSIFFKPPIDIEILFDNEESRKHVDIATRSSNSSYKSMKESLPVYEDGE 49

PfVps26     44 DINGTATISLK-PGKKFEHYGIKLELIGQINILNDKAN-------------- 80
HsVps26a    47 SVSGKVNLAFKQPGKRLEHQGIRIEFVGQIELFNDKSN-------------- 84
ScVps26     50 SLGGIVTLRVR-DSKKVDHLGIKVSVIGSIDMLKSHGSGNSSSKKVTSSTSS 100

PfVps26     81 -------------SYDFFSISKDLEPPGFLVESKQFKWKFSAVDKQHESYFG 119
HsVps26a    85 -------------THEFVNLVKELALPGELTQSRSYDFEFMQVEKPYESYIG 123
ScVps26    101 SSSNGSVDVRKNSVDQFLCQSYDLCPAGELQHSQSFPFLFRDLSKRYESYKG 152

PfVps26    120 TNVQLRYFVRLNIIKGYSGNIQKEID-------------------------- 145
HsVps26a   124 ANVRLRYFLKVTIVRRLT-DLVKEYD-------------------------- 148
ScVps26    153 KNVDVAYYVKVTVMRKST-DISKIKRFWVYLYNSVTTAPNTLSANETKATTN 203

PfVps26    146 ------------------------FI-VQNLCIPPEINN---TIKMEVGIED 169
HsVps26a   149 ------------------------LI-VHQLATYPDVNN---SIKMEVGIED 172
ScVps26    204 DIAGGNYAADNASDNTQTKSTQGEAADVNQVLPISHSNNEPKPVRLDIGIEN 255

PfVps26    170 CLHIEFEYDKSKYHLKDVVVGKVYFLLVRIKIKHMELDIIKMETSGVGKNYT 221
HsVps26a   173 CLHIEFEYNKSKYHLKDVIVGKIYFLLVRIKIQHMELQLIKKEITGIGPSTT 224
ScVps26    256 CLHIEFEYAKSQYSLKEVIVGRIYFLLTRLRIKHMELSLITRESSGLQTSNV 307

PfVps26    222 TETVTLSKFEIMDGSPIKSECIPVRLYLSGFDLTPTYKNIQNKFSVKYYINL 273
HsVps26a   225 TETETIAKYEIMDGAPVKGESIPIRLFLAGYDPTPTMRDVNKKFSVRYFLNL 276
ScVps26    308 MTDSTAIRYEIMDGSSVKGETIPIRLFLSGYDLTPNMS--CNYFNVKNYLSL 357

PfVps26    274 IIVDEEERRYFKKQEIFLWRKKMG                             297
HsVps26a   277 VLVDEEDRRYFKQQEIILWRKAPEKLRKQRTNFHQRFESPESQASAEQPEM  327
ScVps26    358 VIIDEDGRRYFKQSEITLYRTR                               379
```

Figure S1. Sequence alignments

C. Vps35

```
PfVps35     1 MSTYKDNNNHNNHNSNMNTIDQKKFLDECIFVVKEQSFYMKQALENGSLRDT 52
HsVps35     1 MPTTQQSPQD----------EQEKLLDEAIQAVKVQSFQMKRCLDKNKLMD 42
ScVps35     1 MAYADSPE-------------------NAIAVIKQRTALMNRCLSQHKLMES 33

PfVps35    53 LKYASNMLCELRTSHLSPKYYYELYMLIFNELQHLDNFISDKKKHKKKFIDI 104
HsVps35    43 LKHASNMLGELRTSMLSPKSYYELYMAISDELHYLEVYLTDEFAKGRKVADL 94
ScVps35    34 LQHTSIMLTELRNPNLSPKKYYELYVIIFDSLTNLSTYLIENHPQNHHLADL 85

PfVps35   105 YESVQHAGNIIPRLYLLIIVGRNYIKNKDIKAKYILKDMTELCKGVQHPLRG 156
HsVps35    95 YELVQYAGNIIPRLYLLITVGVVYVKSFPQSRKDILKDLVEMCRGVQHPLRG 146
ScVps35    86 YELVQYTGNVVPRLYLMITVGTSYLTFNEAPKKEILKDMIEMCRGVQNPIRG 137

PfVps35   157 LFLRYFLIQMCKDRIPDTGSEYEEAGGGDINDAFEFLLTNFYESLKLWSRMN 208
HsVps35   147 LFLRNYLLQCTRNILPDEGEPTDEETTGDISDSMDFVLLNFAEMNKLWVRM- 197
ScVps35   138 LFLRYYLSQRTKELLPED----------DPSFNSQFIMNNFIEMNKLWVRL- 178

PfVps35   209 DKVLKVPNMIQDDNTMNSKIKILKEKMDVKMLVGSILVRMSQLEGMTKQYYI 260
HsVps35   198 ----------QHQGHSRDREKRERERQELRILVGTNLVRLSQLEGVNVERYK 239
ScVps35   179 ----------QHQGPLRERETRTRERKELQILVGSQLVRLSQIIDDNFQMYK 220

PfVps35   261 ENCLPKILLYLSNINDCLIQQYIFESIVQVFSDECHIYSLEILLNAILKMNT 312
HsVps35   240 QIVLTGILEQVVNCRDALAQEYLMECIIQVFPDEFHLQTLNPFLRACAELHQ 291
ScVps35   221 QDILPTILEQVIQCRDLVSQEYLLDVICQVFADEFHLKTLDTLLQTTLHLNP 272

PfVps35   313 SIDFKSILITLLKRLRSFIEANNKCD-------LPKDIDIFNLFYDHLVVYV 357
HsVps35   292 NVNVKNIIIALIDRLALFAHREDGPG-------IPADIKLFDIFSQQVATVI 336
ScVps35   273 DVSINKIVLTLVDRLNDYVTRQLEDDPNATSTNAYLDMDVFGTFWDYLTVLN 324

PfVps35   358 NRTLDTYTKVNYNDSINRSPNEDHHGYTHNDLSKGKAKDIINNNDNNNDDNN 409
HsVps35   337 QSRQD----------------------------------------------- 341
ScVps35   325 HERPD----------------------------------------------- 329

PfVps35   410 NNKDNDDNNNNNNNNNNNNNNNNNYNYNHNNNQDRYNISKGNVQNGHIKIEN 461
HsVps35       ----------------------------------------------------
ScVps35       ----------------------------------------------------

PfVps35   462 MNQTNNDLNNNNIINNMGHINTNDLNKNNTGKNCTIVNTDEFVNNVVKMLQV 513
HsVps35   342 -------------------------------------------MPSEDVVSL 350
ScVps35   330 -------------------------------------------LSLQQFIPL 338

PfVps35   514 IYEFIFLCIRIYDDDI-IISKLFGLPYTIVSNVNMNNDTICEEIISIIVLPF 564
HsVps35   351 QVSLINLAMKCYPDRVDYVDKVLETTVEIFNKLNLEHIATSS---------- 392
ScVps35   339 VESVIVLSLKWYPNNFDNLNKLFELVLQKTKDYGQKNISLESEHLFLVLLSF 390

PfVps35   565 NYLGLSALNARNMQTLLNSITEKHKKKLSLDIIDAIIECKKKYITYEDVEKI 616
HsVps35   393 ------AVSKELTRLLKIPVDTYNNILTVLK--LKHFHPLFEYFDYESRKSM 436
ScVps35   391 QNSKL-QLTSSTTAPPNSPVTSKKHFIFQLISQCQAYKNILALQSISLQKKV 441

PfVps35   617 LKYISYIFHEK-DKKNKNNDE-------------------DIFNLENNNSAY 648
HsVps35   437 SCYVLSNVLDYNTEIVSQDQ---------------------VDSIMNLVSTL 467
ScVps35   442 VNEIIDILMDREVEEMADNDSESKLHPPGHSAYLVIEDKLQVQRLLSICEPL 493

PfVps35   649 TCEKICKFFHIITNTKNIEEKYNICMLFYKYISNSTYLVHLLPTIIFTLLHV 700
HsVps35   468 IQDQPDQPVEDPDPEDFADE----------------------QSLVGRFIHL 497
ScVps35   494 IISRSGPPANVASSDTNVDEVF-----FNRHDEEESWILDPIQEKLAHLIHW 540
```

Figure S1. Sequence alignments

```
PfVps35     701 VTQITKLGQEDHFNQNDDKNNNMNTIDTIDSNNNNNDNNSNCNNDNSNIDSN 752
HsVps35     498 LRSEDP-------------DQQYLILNTARKHFGAGGN-------------- 522
ScVps35     541 IMNTTSRKQTMKNKIQFSLEAQLEILLLIKSSFIKGG--------------- 577

PfVps35     753 HTMDNEKKEDFINSSDSYNIYPNDNDKQNLNNYLHNNQNNFILDEKKINQYN 804
HsVps35     523 ------QRIRFTLPPLVFAAYQLAFRYK--------------ENSKVDDKWE 554
ScVps35     578 ------INVKYTFPAIITNFWKLMRKCRMIQEYLLKKRP---DNKTLLSHYS 620

PfVps35     805 IYVKNIFKFIHTNLLTVAS----QMPILTFKLFLYSAIVVNNYNSFVQTHEF 852
HsVps35     555 KKCQKIFSFAHQTISALIK---AELAELPLRLFLQGALAAG----------E 593
ScVps35     621 NLLKQMFKFVSRCINDIFNSCNNSCTDLILKLNLQCAILAD----------Q 662

PfVps35     853 LTFDNLEAICYEFITQPLIIYEEDINISAQQFDCIVWIVGILCTHINLLDNE 904
HsVps35     594 IGFENHETVAYEFMSQAFSLYEDEISDSKAQLAAITLIIGTFERMK-CFSEE 644
ScVps35     663 LQLN---EISYDFFSQAFTIFEESLSDSKTQLQALIYIAQSLQKTRSLYKE  711

PfVps35     905 NYNNIALKLTQHANKLLKKKDQCIGVLKCSHLYW-------------ENKKY 943
HsVps35     645 NHEPLRTQCALAASKLLKKPDQGRAVSTCAHLFW------SGRNTDKNGEEL 690
ScVps35     712 YYDSLIVRCTLHGSKLLKKQDQCRAVYLCSHLWWATEISNIGEEEGITDNFY 763

PfVps35     944 RNSNKVIECLQKSIKNAEIAIQSNNDNIILFTYMLDKYLYYYEAQN---IDV 992
HsVps35     691 HGGKRVMECLKKALKIANQ-CMDPSLQVQLFIEILNRYIYFYEKEN---DAV 738
ScVps35     764 RDGKRVLECLQRSLRVADS-IMDNEQSCELMVEILNRCLYYFIHGDESETHI 814

PfVps35     993 SEETLHYLIDICQDYYNKT--NDDTN-------------------------- 1016
HsVps35     739 TIQVLNQLIQKIREDLPNLESSEETEQ------------------------- 765
ScVps35     815 SIKYINGLIELIKTNLKSLKLEDNSASMITNSISDLHITGENNVKASSNADD 866

PfVps35    1017 -------------------------------------------------FKQ 1019
HsVps35         ---------------------------------------------------I 766
ScVps35     867 GSVITDKESNVAIGSDGTYIQLNTLNGSSTLIRGVVATASGSKLLHQLKYIP 918

PfVps35    1020 EYKKVIKYVHDKQKNSNVFQKINIDTSILRS                      1050
HsVps35     767 NKHFHNTLEHLRLRRESPESEGPIYEGLIL                       796
ScVps35     919 IHHFRRTCEYIESQREVDDRFKVIYV                           944
```

D. Rab7

```
                                      ■
PfRab7       1  MSNKKRTILKVIILGDSGVGKTSLMNQYVNKKFTNQYKATIGADFLTKETIVD-N  54
HsRab7a      1  MTSRKKVLLKVIILGDSGVGKTSLMNQYVNKKFSNQYKATIGADFLTKEVMVD-D  54
Ypt7p        1  MSSRKKNILKVIILGDSGVGKTSLMHRYVNDKYSQQYKATIGADFLTKEVTVDGD  55

                         ●
PfRab7      55  EQITMQIWDTAGQERFQSLGVAFYRGADCCVLVFDLTNYKTYESLESWKDEFLIQ  109
HsRab7a     55  RLVTMQIWDTAGQERFQSLGVAFYRGADCCVLVFDVTAPNTFKTLDSWRDEFLIQ  109
Ypt7p       56  KVATMQVWDTAGQERFQSLGVAFYRGADCCVLVYDVTNASSFENIKSWRDEFLVH  110

                           ★
PfRab7     110  ASPKDPENFPFVIIGNKVDET-NKRKVQSLKVLQWCKSNNNIPYFETSAKNAINV  163
HsRab7a    110  ASPRDPENFPFVVLGNKIDLE-N-RQVATKRAQAWCYSKNNIPYFETSAKEAINV  162
Ypt7p      111  ANVNSPETFPFVILGNKIDAEESKKIVSEKSAQELAKSLGDIPLFLTSAKNAINV  165

PfRab7     164  DQAFDEIARKAMKQEHQEEQIY--LPETFALNNQSEQKMYKSRCC           206
HsRab7a    163  EQAFQTIARNALKQE-TEVELYNEFPEPIKLDKNDRAKASAESCSC          207
Ypt7p      166  DTAFEEIARSALQQNQADTEAF---EDDYNDAINIRLDGENNSCSC          208
```
