# Supplementary material for: Evidence for a Golgi-to-Endosome Protein Sorting Pathway in Plasmodium falciparum
Source: PLoS One. 2014 Feb 25;9(2):e89771. doi: 10.1371/journal.pone.0089771 (PMC3934947; doi:10.1371/journal.pone.0089771)

Figure S2. Southern blot characterization of transgenic parasite lines.

A. PfVps29-YFP

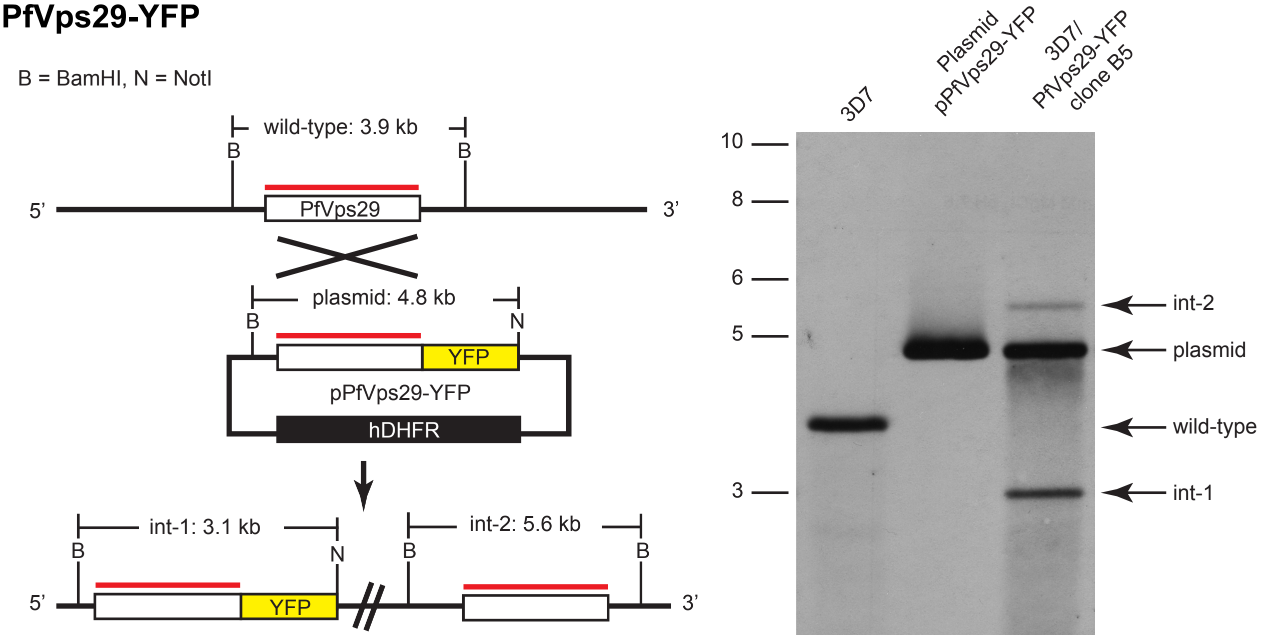

B. PfVps35-YFP

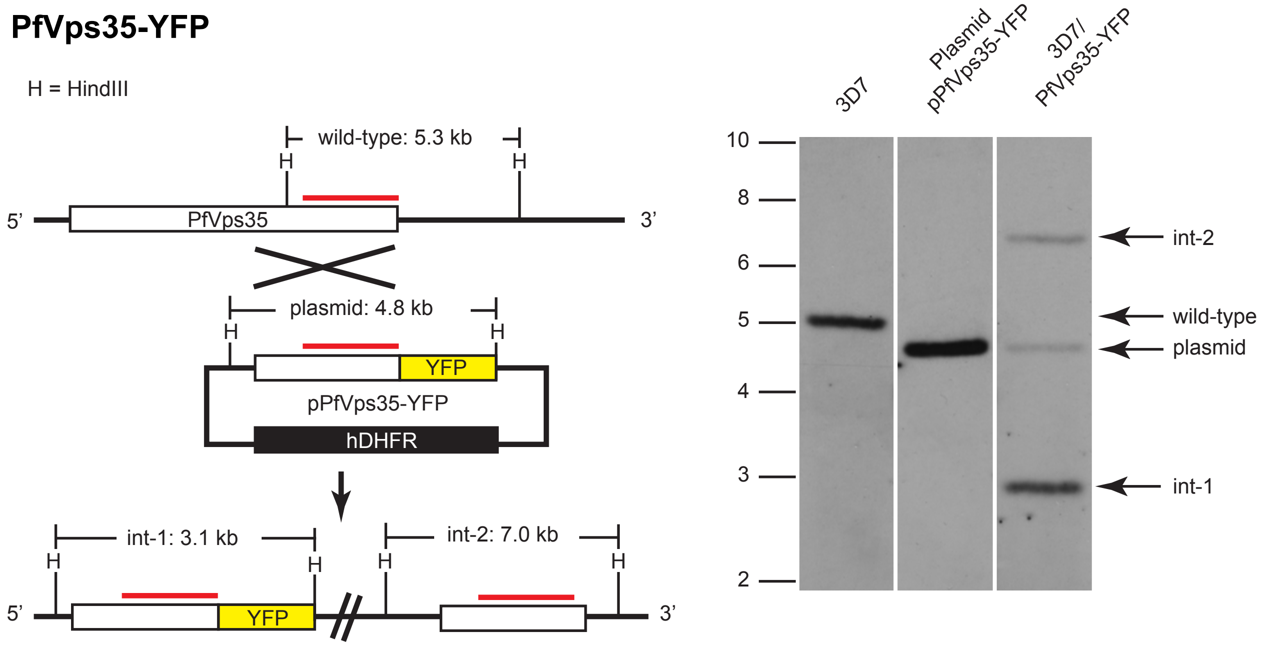

Figure S2. (Continued)

C. PfVps35 gene disruption

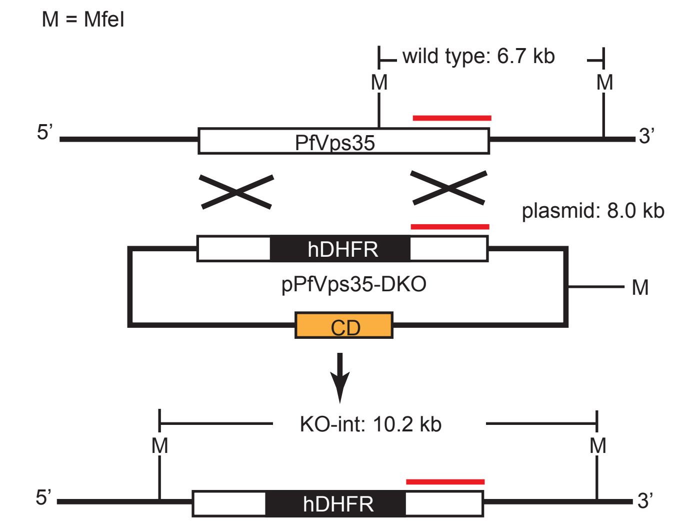

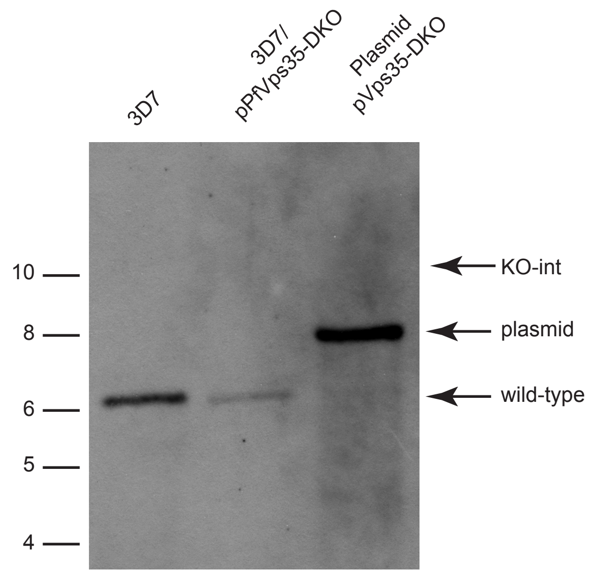

D. DD-mCherry-PfRab7

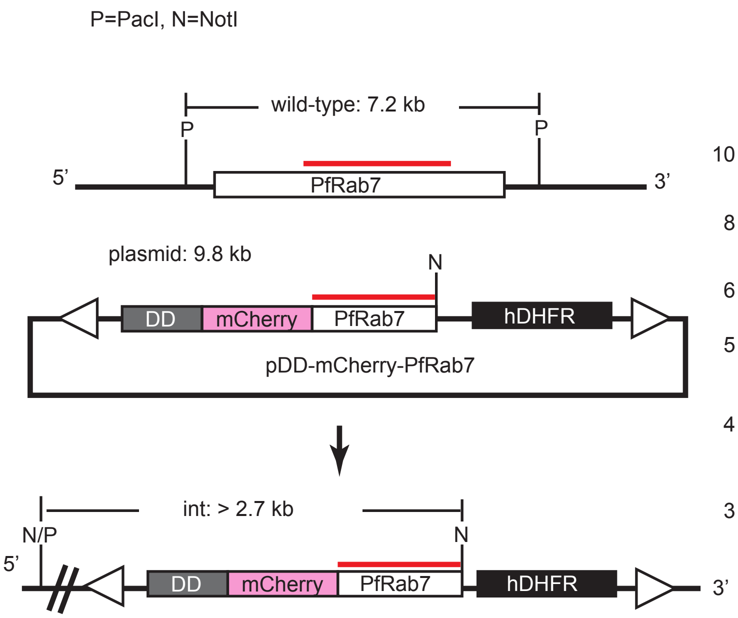

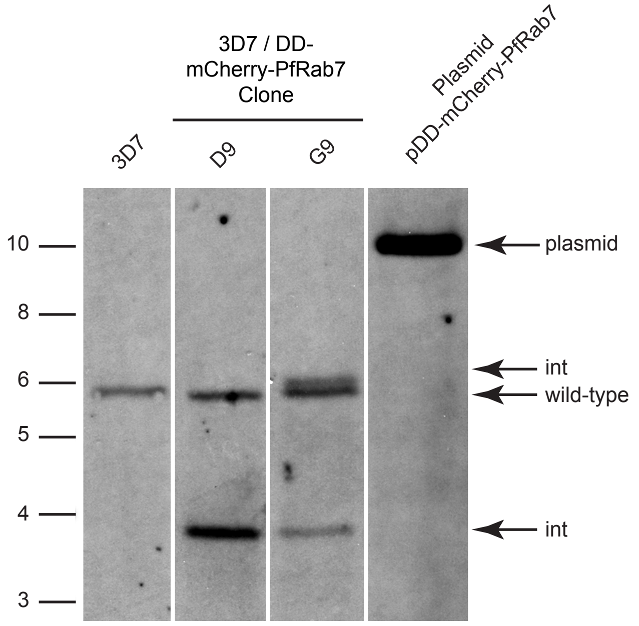

Figure S2. (Continued)

E. PfVps35-HA

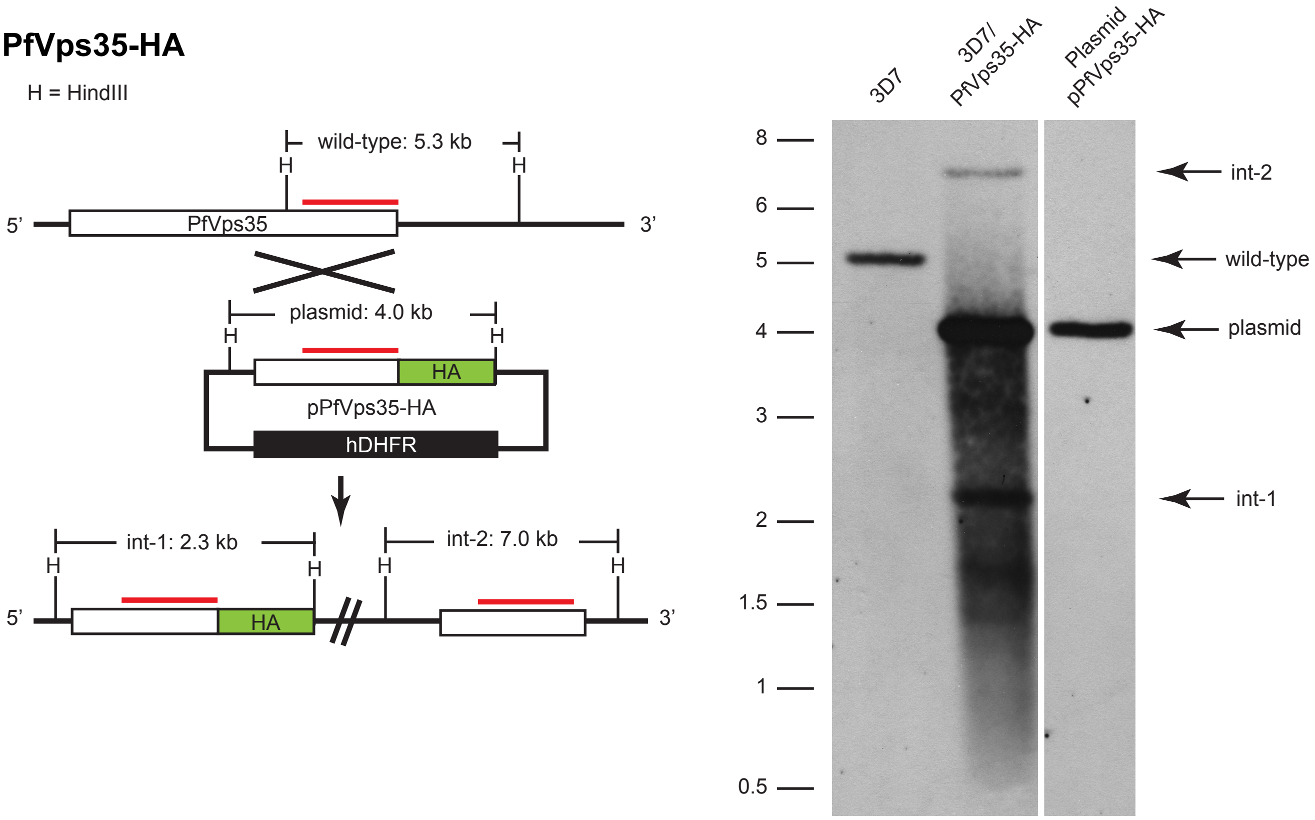

Supplement: Figure S2 — Southern blot characterization of transgenic parasite lines. In each panel, a schematic diagram of the product of recombination or transposition is provided on the left. The predicted sizes of DNA fragments upon digestion with the indicated restriction enzyme are shown. The red bar indicates the position of the probe used for Southern blotting. “X” indicates the expected site(s) of homologous recombination, where applicable. Figures are not drawn to scale. Southern blots of genomic DNA from parental and transfected parasite lines are shown on the right. Each blot was performed on a single membrane. For clarity some lanes are shown as individual strips but all strips originate from the same exposure. Sizes of DNA markers in kilobases are indicated at left. Arrows at right identify the positions of the expected bands. The presence of a “plasmid” band in the transfected parasite lines in A, B and E likely reflects the integration of concatameric episomes. Abbreviations: hDHFR, human dihydrofolate reductase; YFP, yellow fluorescent protein; CD, cytosine deaminase; DD, E. coli DHFR destabilization domain; HA, hemagglutinin epitope tag. (A) Parasite line expressing PfVps29-YFP generated by single-crossover recombination with pPfVps29-YFP. (B) Parasite line expressing PfVps35-YFP generated by single-crossover recombination with pPfVps35-YFP. (C) Parasite line transfected with pPfVps35-DKO and selected for double-crossover disruption of the PfVps35 coding sequence. (D) Two clonal parasite lines (D9 and G9) obtained by transfection with a plasmid (pDD-mCherry-PfRab7) carrying a transposable DD-mCherry-PfRab7 expression cassette. (E) Parasite line expressing PfVps35-HA following single-crossover recombination with pPfVps35-HA. (PDF) [file pone.0089771.s002.pdf]
