# Supplementary figures and images for: Evidence for a Golgi-to-Endosome Protein Sorting Pathway in Plasmodium falciparum
Source: PLoS One. 2014 Feb 25;9(2):e89771. doi: 10.1371/journal.pone.0089771 (PMC3934947; doi:10.1371/journal.pone.0089771)

**Figure S3. Generation of a parasite line expressing PfSortilin-HA.**

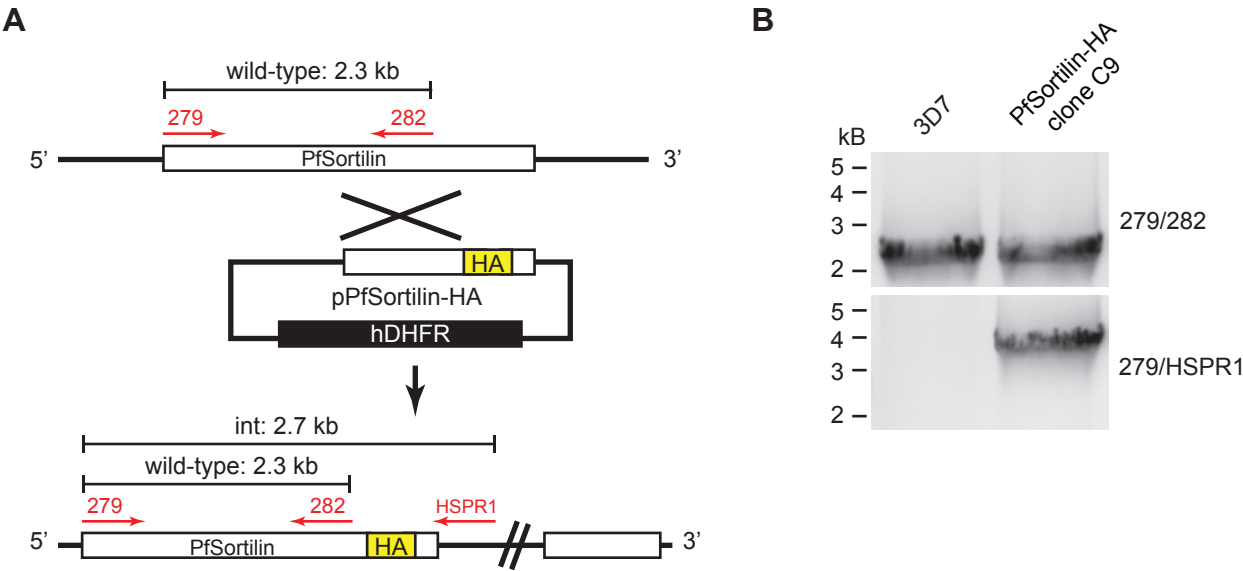

Supplement: Figure S3 — Generation of a parasite line expressing PfSortilin-HA. (A) Schematic diagram of the single-crossover strategy for modifying the PfSortilin chromosomal locus to incorporate an internal hemagglutinin (HA) tag (indicated by the yellow box). Primers used for PCR analysis are indicated with red arrows and predicted sizes of amplified regions are shown. The figure is not drawn to scale. hDHFR, human dihydrofolate reductase. (B) PCR analysis of genomic DNA obtained from clonal parasite line C9 expressing PfSortilin-HA and from the parental 3D7 line using primers indicated in (A). Sizes of DNA markers are shown at left. The presence of a band with primers 279/HSPR1 (lower panel) is consistent with the expected integration event in clone C9. The primer pair 279/282 serves as a PCR control. (PDF) [file pone.0089771.s003.pdf]
