# Supplementary material for: Evidence for a Golgi-to-Endosome Protein Sorting Pathway in Plasmodium falciparum
Source: PLoS One. 2014 Feb 25;9(2):e89771. doi: 10.1371/journal.pone.0089771 (PMC3934947; doi:10.1371/journal.pone.0089771)
Supplement: Table S1 — Sequences of oligonucleotides used in this study. (PDF) [file pone.0089771.s004.pdf]

Table S1: Sequences of oligonucleotides used in this study. Restriction sites are underlined. Where two restriction sites are present, the second is double underlined. RE, restriction endonuclease; UTR, untranslated region; ecDHFR-DD, *E. coli* dihydrofolate reductase destabilization domain; yDHOD, yeast dihydroorotate dehydrogenase; HA, hemagglutinin epitope tag; PfAPP, *P. falciparum* aminopeptidase P; HSP, heat shock protein.

Name	Oligonucleotide Sequence (5' to 3')	RE site	Purpose
334	GCACGCTCGAGAGTGGGAAATTGGAAGATATTGG	XhoI	PCR: PfVps29 bases 4 to 582
335	GCACGCCTAGGTATAACTGTTGACTTATGTAGTTCAC	AvrII	
352	GCACGCTCGAGATACCAATTTATTAACAGTAGCTAGC	XhoI	PCR: PfVps35 bases 2444 to 3150
353	GCACGCCTAGGTGATCTCAATATGGATGTATCTATATTAATTTTCTG	AvrII	
428	GCACGCTCGAGCTATATCAAAAGATC	XhoI	PCR: PfVps26 bases 257 to 891
429	GCACGCCTAGGACCCATTTTTTTTCG	AvrII	
344	GTACGCCGCGGAAAATATGCCTCAAATATGCTGTGC	SacII	PCR: PfVps35 bases 368 to 938
345	GTACGACTAGTAGATCCAACTAACATTTTTACATCC	SpeI	
346	GTACGCCATGGTTATTAACAGTAGCTAGCCAAATGC	NcoI	PCR: PfVps35 bases 2661 to 3238
347	GTACGCCTAGGTTATTGTAATAATCTTGGCATATGTC	AvrII	
456	GCACGCTCGAGCCGTGTTATTATTTAAAGATGAGTGG	XhoI	PCR: PfVps29 bases 1 to 582
457	GTACGCCTAGGTATAACTGTTGACTTATGTAGTTCAC	AvrII	
458	GCACGCCCGGGTCTATTTTCTGTTTTCAATTTTTCACAC	XmaI	PCR: PfVps29 5' UTR (-800 to -20)
459	CACGGCTCGAGAAAAAAAAAAAAAAATATATGAAAATA	XhoI	
460	GTACGCCTAGGGAAAATTTATATTTTCAAAGTGTGAGCAAGGGCGAGGAGGATAAC	AvrII	PCR: mCherry bases 4 to 711
461	GTACGGCGGCCGCTTACTTGTACAGCTCGTCCATGC	NotI	
470	GTACGCTCGAGCTTAAAAGAATAATAATGATCAGTCTGATTGCGGCGTTAG	XhoI	PCR: ecDHFR-DD bases 1 to 474
471	GTACGCCTAGGTCCTCCTCCTCCTCCCCGCCGCTCCAGAATCTCAAAGC	AvrII	
484	GTACGCCTAGGGTGAGCAAGGGCGAGGAGGATAAC	AvrII	PCR: mCherry bases 4 to 708
485	GTACGGCGGCCGCTACTCCGCGGACTTTGAAAATATAAATTTTCCTTGTACAGCTCGTCCAT GCCGC	SacII/NotI	
476	GTACGCCCGGGAATAAAACATTTTAATTGCACATGTGTG	XmaI	PCR: PfRab7 5' UTR (-880 to -17)
477	GTACGCTCGAGTTGTTCTTTTTTTTTTTTTTTTTTTATTAAAATTTGATTTTAC	XhoI	
478	GTACGCCGCGGTCAAATAAAAAAAGAACCATATTAAAAG	SacII	PCR: PfRab7 bases 4 to 621
479	GTACGGCGGCCGCTTAACAACAACGACTTTTGTACATTTTTTG	NotI	
480	GTACGCCCGGGTTTCTTTTGTTTGAATTTAAAATATTTTAGAG	XmaI	PCR: PfRab6 5' UTR (-806 to -16)
481	GTACGCTCGAGAAAATAAAAAAATATTTTTAATTAAGAATTAGTC	XhoI	

482	GTACGCCGCGGGATGAATTTCAAAACTCGGGAC	SacII	PCR: PfRab6 bases 4 to 624
483	GTACGGCGGCCGCTTAACATAAACATTTACTTAACATATTTTTG	NotI	
597	GTACGGCGGCCGCTTACTTGTACAGCTCGTCCATGC	NotI	PCR: mCherry with 3' stop codon
598	GTACGGCGGCCGCTTAACGACTTTTGTACATTTTTTGTTCG	NotI	PCR: PfRab7ΔCC bases 4 to 612
599	GTACGAGATCTTACTCCGCGGTCAAATAAAAAAAGAACCATATTAAAAG	BglII/SacII	PCR: PfRab7 transfer to pSP72
600	GTACGCTCGAGTACTGCGGCCGCTTAACAACAACGACTTTTGTACATTTTTTG	XhoI/NotI	
591	CTTGGAGATAGTGGTGTTGGTAAAAATTCATTAATGAATCAATATGTG	none	Quikchange: PfRab7 T22N
592	CACATATTGATTCATTAATGAATTTTTACCAACACCACTATCTCCAAG	none	
593	GATATGGGATACTGCAGGACTAGAACGTTTTCAAAGTTTAGGAG	none	Quikchange: PfRab7 Q67L
594	CTCCTAAACTTTGAAAACGTTCTAGTCCTGCAGTATCCCATATC	none	
595	GAAAATTTTCCTTTTGTTATTATTGGACTTAAAGTTGATGAAACAAATAAAAG	none	Quikchange: PfRab7 N125I
596	CTTTTATTTGTTTCATCAACTTTAAGTCCAATAATAACAAAAGGAAAATTTTC	none	
251	GTACGCCCGGGTAAGTTTTTTTTATTTGATATAGAAAC	XmaI	PCR: PfAPP 5’ UTR (-865 to -39)
144	GCACGCTCGAGATTTAAAAAAAAAAAAAGAAAGAAGAAG	XhoI	
HSPR1	TATATATGTATATTGGGGTGATG	none	Sequencing: HSP86 3’ UTR reverse
276	GCACGCTCGAGGTCAATATTATTTATAATATTTATCATGG	XhoI	PCR: PfSortilin bases 2476 to 3106
283	GTACGCCTAGGTGAAGGTGATATATTCCTTGAGGT	AvrII	
286	CTAGGTATCCATATGATGTACCAGATTATGCAAAAAATTATGCAGATAATATTGAATTATTA TAAGC	AvrII/NotI	Annealing oligos: HA tag and PfSortilin bases 3107 to 3139
287	GGCCGCTTATAATAATTCAATATTATCTGCATAATTTTTTGCATAATCTGGTACATCATATGG ATAC	AvrII/NotI	
501	CTACGAGATCTCATTTTGTAAAAAAAATTAAAATATATTTATAT	BglII	PCR: yDHOD cassette
502	CTACGGAATTCTTTAATAAATATGTTCTTATATATAATGAG	EcoRI	
245	TCGAAGGCGCCATATACCCGGGATATAC	KasI/XmaI	Annealing oligos: pXL-BacII linker
246	TCGAGTATATCCCGGGTATATGGCGCCT	XmaI/KasI	
