# Supplementary material for: Evidence for a Golgi-to-Endosome Protein Sorting Pathway in Plasmodium falciparum
Source: PLoS One. 2014 Feb 25;9(2):e89771. doi: 10.1371/journal.pone.0089771 (PMC3934947; doi:10.1371/journal.pone.0089771)
Supplement: Table S2 — Plasmids generated in this study. (PDF) [file pone.0089771.s005.pdf]

Table S2: Plasmids generated in this study. YFP, yellow fluorescent protein; DD, *E. coli* dihydrofolate reductase destabilization domain; HA, hemagglutinin tag; hDHFR, human dihydrofolate reductase; yDHOD, yeast dihydroorotate dehydrogenase.

A. Plasmids for chromosomal integration by homologous recombination.

Protein	Modification	Resistance	Name
PfVps29	C-terminal YFP	hDHFR	pPfVps29-YFP
PfVps26	C-terminal YFP	hDHFR	pPfVps26-YFP
PfVps35	C-terminal YFP	hDHFR	pPfVps35-YFP
	C-terminal HA	hDHFR	pPfVps35-HA
	Double cross-over recombination for gene disruption	hDHFR	pPfVps35-DKO
PfSortilin	Internal HA tag	hDHFR	pSortilin-HA

B. Plasmids carrying *piggyBac* transposable expression cassettes.

Gene	Cassette organization (*promoter*/coding sequences)	Resistance	Name
PfVps29	*pfvps29*/PfVps29-mCherry	hDHFR	pPfVps29-mCherry
	pfvps29/PfVps29-mCherry	yDHOD	pPfVps29-mCherry-yDHOD
PfRab7	*pfrab7*/DD-mCherry	hDHFR	pDD-mCherry
	pfrab7/DD-mCherry-PfRab7	hDHFR	pDD-mCherry-PfRab7
	pfrab7/DD-mCherry-PfRab7	yDHOD	pDD-mCherry-PfRab7-yDHOD
	pfrab7/DD-mCherry-PfRab7(C-terminal 2-residue truncation)	hDHFR	pDD-mCherry-PfRab7ΔCC
	pfrab7/DD-mCherry-PfRab7 T22N	hDHFR	pDD-mCherry-PfRab7T22N
	pfrab7/DD-mCherry-PfRab7 N125I	hDHFR	pDD-mCherry-PfRab7N125I
	pfrab7/DD-mCherry-PfRab7 Q67L	hDHFR	pDD-mCherry-PfRab7Q67L
	pfapp/DD-mCherry-PfRab7 T22N	hDHFR	pPfAPPp-DD-mCherry-PfRab7T22N
	pfapp/DD-mCherry-PfRab7 N125I	hDHFR	pPfAPPp-DD-mCherry-PfRab7N125I
	pfapp/DD-mCherry-PfRab7 Q67L	hDHFR	pPfAPPp-DD-mCherry-PfRab7Q67L
PfRab6	*pfrab6*/DD-mCherry-PfRab6	hDHFR	pDD-mCherry-PfRab6
	pfrab6/DD-mCherry-PfRab6	yDHOD	pDD-mCherry-PfRab6-yDHOD
